# Perturbation of Ephrin Receptor Signaling and Glutamatergic Transmission in the Hypothalamus in Depression Using Proteomics Integrated With Metabolomics

**DOI:** 10.3389/fnins.2019.01359

**Published:** 2019-12-17

**Authors:** Yu Wu, Zhenhong Wei, Yonghong Li, Chaojun Wei, Yuanting Li, Pengfei Cheng, Hui Xu, Zhenhao Li, Rui Guo, Xiaoming Qi, Jing Jia, Yanjuan Jia, Wanxia Wang, Xiaoling Gao

**Affiliations:** ^1^The Institute of Clinical Research and Translational Medicine, Gansu Provincial Hospital, Lanzhou, China; ^2^NHC Key Laboratory of Diagnosis and Therapy of Gastrointestinal Tumor, Gansu Provincial Hospital, Lanzhou, China; ^3^Gansu Provincial Biobank and Bioinformation Engineering Research Center, Lanzhou, China; ^4^Department of Neurology, The First Affiliated Hospital of Jiamusi University, Jiamusi, China

**Keywords:** depression, inflammation, hypothalamus, omics, Ephrin receptor signaling, glutamatergic transmission

## Abstract

Hypothalamic dysfunction is a key pathological factor in inflammation-associated depression. In the present study, isobaric tags for relative-absolute quantitation (iTRAQ) combined with mass spectrometry and gas chromatography-mass spectrometry (GC-MS) were employed to detect the proteomes and metabolomes in the hypothalamus of the lipopolysaccharide (LPS)-induced depression mouse, respectively. A total of 187 proteins and 27 metabolites were differentially expressed compared with the control group. Following the integration of bi-omics data, pertinent pathways and molecular interaction networks were further identified. The results indicated altered molecules were clustered into Ephrin receptor signaling, glutamatergic transmission, and inflammation-related signaling included the LXR/RXR activation, FXR/RXR activation, and acute phase response signaling. First discovered in the hypothalamus, Ephrin receptor signaling regulates *N*-methyl-D-aspartate receptor (NMDAR)-predominant glutamatergic transmission, and further acted on AKT signaling that contributed to changes in hypothalamic neuroplasticity. Ephrin type-B receptor 2 (EPHB2), a transmembrane receptor protein in Ephrin receptor signaling, was significantly elevated and interacted with the accumulated NMDAR subunit GluN2A in the hypothalamus. Additionally, molecules involved in synaptic plasticity regulation, such as hypothalamic postsynaptic density protein-95 (PSD-95), p-AKT and brain-derived neurotrophic factor (BDNF), were significantly altered in the LPS-induced depressed group. It might be an underlying pathogenesis that the EPHB2-GluN2A-AKT cascade regulates synaptic plasticity in depression. EPHB2 can be a potential therapeutic target in the correction of glutamatergic transmission dysfunction. In summary, our findings point to the previously undiscovered molecular underpinnings of the pathophysiology in the hypothalamus of inflammation-associated depression and offer potential targets to develop antidepressants.

## Introduction

Depression, characterized by depressed mood, diminished interests, avolition, worthlessness and hopelessness, raises concern in global public health (Otte et al., 2016). The lifetime prevalence of depression is estimated at 2–20%, and more than 60% of depression cases jeopardize the quality of life (Zilcha-Mano et al., 2014; Ribeiro et al., 2018). Depression is a multifactorial and heterogeneous disorder with abundant etiologies, molecular alterations and multiple pathway dysregulations. Among the currently known pathogenetic factors, inflammation has been considered an important pathologic factor of depressive disorders in vulnerable individuals, such as patients with systemic infections, diabetes, cancers, or autoimmune diseases, *etc*. (Dantzer et al., 2008; Maes and Carvalho, 2018). Increasing evidence has highlighted several mental illnesses including depression have been linked to HPA axis dysfunctions (Jacobson, 2014; Rao et al., 2016). However, regulating the function of the HPA axis in inflammation-associated depression remains poorly understood.

The hypothalamus, a vital neuroendocrine region of the HPA axis, is closely related to the pathogenesis of depression (Gotlib et al., 2008). Many preclinical and clinical studies have proven that certain depression characteristics are associated with abnormities of the hypothalamus (Jacobson, 2014). Thus, neuroimaging and *postmortem* brain microscopy studies have shown widespread anatomical changes, volume deficits, and neuron pathologic changes in the hypothalamus of individuals with depression (Bernstein et al., 2019). Some alterations at the cellular level of the hypothalamus, such as perturbation of hypothalamic peptides such as vasopressin, corticotropin-releasing hormone (CRH), as well as regulatory proteins involved in neurotransmitter metabolism, have been found in depression (Rao et al., 2016; Karisetty et al., 2017; Wu et al., 2017; Bernstein et al., 2019). Furthermore, the hypothalamus is closely communicated to the PFC and hippocampus (Savitz et al., 2013; Bernstein et al., 2019), which are widely recognized as main brain regions in the pathogenesis of MDD (Veeraiah et al., 2014; Guida et al., 2018). The aforementioned evidence has indicated that functional abnormalities of the hypothalamus are critical in the pathophysiology of depression. These abnormalities in the hypothalamus may be a consequence of metabolome, proteome and transcriptome modifications. Integrated analysis of the hypothalamic metabolome and proteome will help us further explore the molecular mechanisms of inflammation-associated depression.

To detect this hypothesis, omics technologies, such as metabolomics and proteomics, have been applied as newfangled means to verify molecular profiling, characterize complex biological mechanisms, identify disease biomarkers, and determine pathophysiological processes in various disease states (Arnett and Claas, 2018). Employing omics approaches, some previous reports have performed a series of preclinical (Rao et al., 2016; Zhou et al., 2016; Karisetty et al., 2017) and clinical (Hashimoto, 2018; Preece et al., 2018; Schubert et al., 2018) investigations on depression. To gain insight into the pathogenesis of depression, metabolomics of the PFC and hypothalamus in inflammation-associated depression mouse model were performed in our previous studies (Wu et al., 2016, 2017). We found dysfunctions of amino acid and purine metabolism in the hypothalamus (Wu et al., 2017). However, the biological interpretation of data from a single type of omics study can be a great challenge due to complex biochemical regulation at multiple levels. Thus, integrated analysis of multiple-omics data is conducive to explore potential biological relationships and improve the understanding of entire biological mechanisms (Misra et al., 2018; Schubert et al., 2018).

To investigate the molecular mechanisms of inflammation-associated depression through integration of the hypothalamic metabolome and proteome in the LPS-induced depression model, the gas chromatography-mass spectrometry (GC-MS)-based metabolomic analysis and iTRAQ combined with LC-MS/MS quantitative proteomics analysis of hypothalamus tissues from LPS-induced depressed and control mice was implemented. To identify potential relationships among the differentially expressed proteins and metabolites, IPA software was used to analyze proteomics and metabolomics data of the hypothalamus. Ephrin receptor signaling, glutamatergic transmission and AKT signaling were identified as key pathways correlated with depression. The key proteins involved in these pathways were validated by qRT-PCR and WB. The obtained results were beneficial to confirm authentic biomolecule differences in the hypothalamus and furnish valuable insights into hypothalamus-based metabolic and proteomic changes in inflammation-mediated depression. Better recognition of the underlying mechanisms of depression may pave a way to antidepressant discovery and diagnostic biomarker exploration.

## Materials and Methods

### Animals and Treatments

In total, 80 adult 12-week-old male CD-1 (ICR) mice (SPF grade) weighing 35 – 40 g were obtained from the Beijing Vital River Laboratory Animal Technology Co., Ltd. (Beijing, China). All mice were housed under controlled illumination and environmental conditions for 2-weeks prior to the commencement of experiments. All the mice were singly housed under a light-dark cycle of 12 h at 55 ± 5% relative humidity and 21 – 22°C and were allowed access to food and water *ad libitum*. To minimize circadian rhythm changes and subjective influence on behavioral tests, mice in each group were intermixed during the observation and the observers were blinded to the intervention conditions. Protocols of animal experimentation were reviewed and approved by the Ethics Committee of Gansu Provincial Hospital (Lanzhou, China). In the handling, the procedures and care of all animals were carried out in compliance with the National Institutes of Health Guide for the Care and Use of Laboratory Animals. All efforts were made to minimize the number of animals used and their suffering.

LPS (L-3129, *Escherichia coli* serotype 0127:B8, Sigma-Aldrich, MO, United States) was freshly dissolved in sterile endotoxin-free isotonic saline. An experimental group (LPS group) was administered by intraperitoneal (i.p.) injection at a dose of 0.83 mg/kg. This dose of LPS was chosen because it elicits a proinflammatory cytokine response in the brain, resulting in depressive-like behaviors in mice (O’Connor et al., 2009; Walker et al., 2013). A control group (CON group) was injected (i.p.) with sterile saline.

### Behavioral Testing and Sample Collection

Two weeks before the experiment, the baseline of food and liquid consumption was monitored. Eight mice were excluded because their sucrose preference or BW did not reach the requirement baseline. The remaining mice were submitted to the depression model. Among them, 38 were for omics experiments and 34 for validation including qRT-PCR and WB analysis. Depressive-like behaviors were then assessed for 24 h following the i.p. injection of LPS. All behavioral tests were performed during the first 4 h of the dark phase of the light cycle (8:00–12:00 a.m.) under conditions of dim light and low noise. The time schedule for the LPS-induced depressive-like model procedure was as previously reported (Walker et al., 2013; Wu et al., 2016). The experimental timeline and study design were presented in Supplementary Figure S1.

#### Sucrose Preference Test, Body Weight and Food Intake

The SPT was conducted to evaluate the anhedonic response after LPS injection as described previously (Walker et al., 2013; Wu et al., 2017). The animals were trained for 2-weeks to consume a palatable sucrose solution (2%). The baseline of sucrose preference was recorded thrice during the first week and twice during the last weeks. The SPT was conducted before the TST and after 24 h LPS treatment. Sucrose preference was calculated as the percentage of sucrose solution ingested relative to the total amount of liquid consumed. Additionally, BW and food consumption were evaluated before and 24 h after LPS treatment.

#### Tail Suspension Test and Forced Swimming Test

The FST and TST were performed as previously described (O’Connor et al., 2009; Wu et al., 2016), and were videotaped and quantified using a video-computerized tracking system (SMART, Panlab, Barcelona, Spain) (Zhe et al., 2017). All the mice were transported to a dimly illuminated behavioral room with minimal noise and were left undisturbed for at least 1h before testing.

##### Tail suspension test

Each mouse was suspended by its tail with adhesive tape placed 2 cm from the tail tip in an acoustically and visually isolated suspension box. The test sessions lasted 6 min with the last 5 min scored for immobility. The mice were considered immobile only when they hung passively or stayed completely motionless.

##### Forced swimming test

Each mouse was placed individually in a Plexiglas cylinder (30 cm in height × 15 cm in diameter) filled with 20 cm of water (24 ± 1°C). The duration of immobility in seconds was monitored by a well-trained observer during the last 5 min of the 6-min test. Immobility was defined as being motionless floating in the water, with only movements that were needed for the mouse to keep its head above water.

#### Sample Collection

After behavioral detection, the mice were euthanized after CO_2_ deep anesthesia (Walker et al., 2013). The hypothalamus was separated from the brain, weighed, and rapidly frozen with liquid nitrogen and stored at −80°C for omics, qRT-PCR and WB experiments.

### Metabolomics Analysis and Differential Metabolite Identification

For GC–MS metabolomics analysis, metabolite extracts were prepared from 20 mouse hypothalami (*n* = 10 in the CON group, *n* = 10 in the LPS group). The hypothalamus samples were pre-processed with a precooled methanol–water–chloroform (5: 2: 2, V/V/V) mixed solution and derivatized with methoxamine hydrochloride and *N,O*-bis(trimethylsilyl)-trifluoroacetamide (BSTFA) with 1% trimethylsilyl chloride (TMCS). The derivative was injected into GC-MS for analysis. Details of the derivatization and GC-MS conditions have been described in our previous publication (Wu et al., 2017). Data analysis was performed using the exported NetCdf file format by TagFinder. The peak areas of extracted ions were normalized to the internal standard _*L*_-2-chlorophenylalanine.

By GC-MS metabolomics profiling of hypothalamus, 177 metabolite components were detected and used for multivariate analysis (Wu et al., 2017). Briefly, the normalized data sets were imported into SIMCA-P software (Version 14.1; Umetrics, Umea, Sweden) and a principal component analysis (PCA) was performed to observe sample distributions. To maximize class discrimination, the data were further undergoing the orthogonal partial least-squares discriminant analysis (OPLS-DA), which validated by a 200-iteration permutation test. The VIP (variable importance in the projection) value > 1 in the OPLS-DA model and *P* < 0.05 in the two-tailed Student’s *t*-test was recognized as the significant difference in metabolite expression.

### Proteomics Analysis

#### Protein Extraction, Digestion and iTRAQ Labeling

For iTRAQ proteomics analysis, 18 hypothalamus samples were obtained from the LPS group (*n* = 9) and CON group (*n* = 9), and then each sample was homogenized and dissolved in 800 μL of SDT buffer [4% sodium dodecyl sulfonate (SDS), 0.1 M dithiosuccinol (DTT), 100 mM Tris-HCl, pH 7.6]. The lysates were sonicated and boiled in water for 15 min and then were centrifuged at high speed (40,000 *g*) for 40 min. Using bovine serum albumin (BSA) as the standard, the protein concentrations in the supernatant were quantified using the bicinchoninic acid (BCA) protein assay kit according to the manufacturer’s instructions (Bio-Rad, United States). Next, 20 μg of proteins for each sample were mixed with 5 × loading buffer and boiled for 5 min. The proteins were then separated by 12.5% sodium dodecyl sulfonate-polyacrylamide gel electrophoresis (SDS-PAGE) gel. The protein bands were visualized by Coomassie Blue R-250 staining, which was used to evaluate the sample quality, verify the quantitative accuracy of the BCA assay and perform inter-group parallelism.

To reduce the influence of individual variation on candidate target selection, protein sample pooling is a commonly used strategy in proteomic studies (Diz et al., 2009). The protein samples from three mice (3, 3, 3) were randomly pooled at the ratio of 1:1:1 as a biological sample to avoid erroneous conclusions due to individual variations. Consequently, three biological replications were obtained for each group. Subsequently, the extracted proteins were digested using the filter-aided sample preparation (FASP) procedure (Wisniewski et al., 2009). The peptide solutions after digestion were vacuum-dried prior to the labeling step.

The resulting 100 μg peptide mixture was labeled with the 8-plex iTRAQ reagent according to the manufacturer’s instructions (AB SCIEX, Framingham, United States). Samples from the CON samples were labeled with iTRAQ tags 113, 114 and 115, while the LPS samples were labeled with tags 116, 117, and 118. After 2 h of incubation at room temperature, all six labeled samples were grouped at equal ratios and were vacuum-dried.

#### Peptide Fractionation Using Strong Cation Exchange Chromatography

The labeled peptides were fractionated by SCX chromatography according to previously described methods (Zhou et al., 2016). Briefly, the dried peptide mixture was reconstituted and acidified with buffer A [10 mM KH_2_PO_4_ in 25% acetonitrile (ACN), pH 3.0] and were loaded onto a Polysulfoethyl 4.6 × 100 mm column (5 μm, 200 Å, PolyLC Inc., Columbia, MD, United States) at a flow rate of 1.0 mL/min. The peptides were separated using a suitable gradient elution buffer B (500 mM KCl, 10 mM KH_2_PO_4_ in 25% ACN, pH 3.0) at a steady flow rate of 1.0 mL/min. The elution was monitored by absorbance at 214 nm, and fractions were collected per minute. The eluted peptides were collected and desalted using an offline fraction collector. The resulting fractions were finally desalted on C18 Cartridges (Sigma, St. Louis, MO, United States), concentrated by vacuum centrifugation, and reconstituted in 40 μl of 0.1% formic acid for mass-spectrometric (MS) detection.

#### Liquid Chromatography-Tandem Mass Spectrometry and Data Analysis

The SCX fractions were analyzed using a Q Exactive mass spectrometer that was coupled to an Easy-nanoLC system (Thermo Fisher Scientific, United States). The peptide mixtures were loaded onto a C18-reversed phase column (Thermo Scientific Easy Column, 10 cm long, 75 μm inner diameter, 3 μm; C18-A2) in buffer A (0.1% formic acid) and were separated with a linear gradient of buffer B (84% ACN and 0.1% formic acid) at a flow rate of 300 nL/min. Next, the eluted peptides were injected into the coupled Q-Exactive mass spectrometer, which was operated in the positive ion mode. The mass was acquired over the range of 300 to 1800 mass/charge (*m/z*). Q-Exactive survey scans were acquired at a resolution of 70,000 at *m/z* 200, and the resolution for higher-energy collisional dissociation (HCD) spectra was 17,500 at *m/z* 200, and the maximum ion injection time was fixed at 20 and 60 ms. The dynamic exclusion duration was 40 s. The normalized collision energy was 30 eV, and the underfill ratio, which specifies the minimum percentage of the target value likely to be reached at the maximum fill time, was defined as 0.1%. The instrument was executed with the peptide recognition mode enabled.

MS/MS spectra were searched using the MASCOT search engine (Matrix Science, London, United Kingdom; version 2.2) embedded into Proteome Discoverer 1.4 (Thermo Fisher Scientific, Waltham, MA, United States). Subsequently, the database (UniProt_Mouse_84433_20180105) from UniProt^1^ with 76,417 protein sequences was downloaded. A decoy database search strategy was performed to calculate the false discovery rate (FDR). The search parameters were set as follows: (i) sample type as iTRAQ 8-plex (peptide-labeled); (ii) trypsin as digestion enzyme; (iii) number of missed cleavages, up to 2; (iv) variable modifications of methionine oxidation; (v) fixed modification of carbamidomethyl cysteine; (vi) peptide mass tolerance of ±20 ppm; and (vii) fragment mass tolerance of 0.1 Da. Proteome Discoverer software was used to extract the peak intensity of each expected iTRAQ reporter ion from each analyzed fragmentation spectrum. The quantitative analysis parameters were as follows: the use of only unique peptides; rejection of all quantification values if not all quantification channels are present; normalization on protein median; normalization all peptide ratios by the median protein ratio. The relative quantity of a peptide among the different samples was determined by comparing the intensities of reporter ion signals also present in the MS/MS scan. The results were then transferred to Microsoft Excel for manual data interpretation. To minimize false-positive results, a strict cutoff for protein identification was applied with an FDR less than 1%. The resulting data set was autobias corrected to eliminate any variations imparted due to unequal mixing of the different labeled samples. This correction assumed that the expression of most proteins did not change. This bias correction mitigates the systematic error arising from samples from each experimental condition that was not combined in exactly equal amounts. The software identified the median average protein ratio, corrected it to unity and then applied this factor to all quantification results (Zhou et al., 2016). Protein identification was supported by all peptide matches with 95% confidence. Compared with CON, the proteins considered to be differentially expressed (or dysregulated) and used for further analysis were filtered with *P*-values < 0.05 and 1.20-fold changes (>1.20 or <0.83), as adopted in previous studies (Zhang et al., 2014; Zhou et al., 2016).

### Bioinformatics

To obtain the functional classification and biological properties of the differentially abundant proteins, the identified protein sequences were mapped using Gene Ontology (GO) terms. Functional annotation tools DAVID 6.8^2^ were adopted to GO functional annotation and functional enrichment of proteins. Three annotated categories, biological process (BP), cellular component (CC) and molecular function (MF), were visualized using R package clusterProfiler^3^. IPA^4^ (Qiagen, Valencia, CA, United States) software was performed to search for biological functions, canonical pathways, and molecular interacted networks of differential metabolites and proteins (Dong et al., 2012). Network analysis can provide a quick solution to evaluate the data of interest regulatory networks, metabolic pathways, and physiological processes. The hypothalamic differential metabolites and proteins results from both groups were submitted to the IPA database. Fisher’s exact test was used in these analyses to identify overexpression of molecular groups. Additionally, other databases, including the Reactome Knowledgebase^5^ (Fabregat et al., 2018), Integrated Molecular Pathway Level Analysis (IMPaLA^6^) (Kamburov et al., 2011), KEGG^7^, and Protein Analysis Through Evolutionary Relationships (PANTHER) pathway^8^ (Mi and Thomas, 2009), were further adopted as a bioinformatics analysis reference to acquire referential value and more detailed information.

### Quantitative Real-Time PCR

qRT-PCR was used to detect the gene expression in the glutamatergic transmission pathway and Ephrin receptor signaling. Ten hypothalamic samples RNA (*n* = 5 mice/group) were extracted in the TRIzol extraction protocol (Life Technologies, Carlsbad, CA, United States) according to the manufacturer’s instructions. cDNA synthesis was performed using the QuantiNova^TM^ reverse transcription kit (QIAGEN). Subsequently, qRT-PCR was performed using the ABI ViiA 7 RT-PCR System (Applied Biosystems, Foster City, CA, United States). A SYBR green detection system (QIAGEN) was used in reactions. The PCR amplification protocol was as follows: initial DNA polymerase activation at 95°C for 2 min, followed by 40 cycles with denaturation at 95°C for 5 s, and annealing extension at 60°C for 10 s. The 2^–ΔΔ*CT*^ method was applied to calculate the relative changes in gene expression using β-Actin values for normalization. Specific primers were obtained from Sangon Biotech (Shanghai, China) and are listed in Supplementary Table S1.

### Western Blotting

Western blotting analysis was performed to identify and validate the proteomics and bioinformatics predicted findings as described previously (Cheng et al., 2015). Mouse hypothalamus samples from both the LPS and CON groups (*n* = 4/group) were homogenized in RIPA buffer followed by ultrasonication on ice. The supernatants were collected and the total protein concentration measured using the BCA assay. Thereafter, equal amounts of protein were separated using 10% SDS-PAGE gels, followed by transfer to PVDF membranes (Millipore, Billerica, MA, United States) for blocking. Next, the membranes were reacted with anti- EFNB1 (diluted 1:1000; Abcam, United Kingdom) and its receptor EPHB2, anti-AKT (diluted 1:2000; Abcam), anti-phospho-AKT (diluted 1:2000; Abcam), anti-glutamine synthetase (Glul, diluted 1:10000; Abcam), anti-glutamate receptor ionotropic-NMDA 1 (GluN1, diluted 1:1000; ABclonal), anti-GluN2A (diluted 1:4000; ABclonal), and anti-GluN2B (diluted 1:1000; ABclonal), anti-postsynaptic density protein-95 (PSD-95, diluted 1:2000; Abcam), and anti-BDNF (diluted 1:2000; Abcam) over night at 4°C. Next, all the membranes were incubated with appropriate secondary antibodies (Bio-Rad, United States) for 2 h at room temperature. Finally, an ECL kit (Millipore, United States) was utilized to visualize signals.

### Statistical Analysis

The data (for behaviors and molecules in validation experiment) were expressed as the means ± standard error of the means (SEM) between two groups. Two-tailed Student’s *t*-test was utilized to analyze the significant difference between the two groups. Statistical analyses were conducted using SPSS 21.0 (SPSS; IBM, Armonk, NY, United States) and GraphPad Prism 6.0 software. A *P*-value < 0.05 was considered statistically significant.

## Results

### Evaluation of the LPS-Induced Depressive-Like Model and Inflammation in the Hypothalamus

**FIGURE 1:**
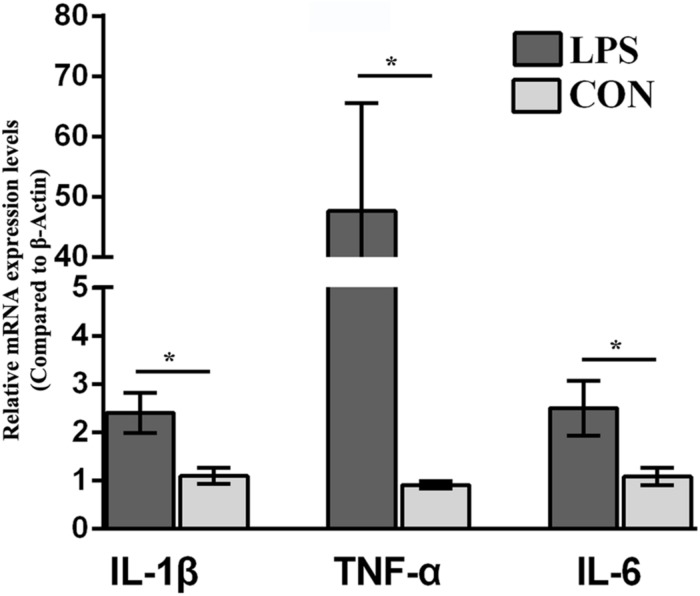
Effect of lipopolysaccharide (LPS, 0.83 mg/kg, i.p.) on the IL-1β, TNF-α, and IL-6 mRNA levels in the hypothalamus. The mice were injected with LPS (0.83 mg/kg, i.p.) and evaluated for behavior 24 h.p.i. Ten hypothalamus samples were collected for proinflammatory cytokine (IL-1β, TNF-α, and IL-6) mRNA analysis (*n* = 5/group). Student’s *t*-test was utilized to analyze the significant difference of the IL-1β, TNF-α, and IL-6 mRNA levels between two groups. The values are presented as the means ± SEM. ^∗^*P* < 0.05, LPS vs. CON.

CD-1 mice were challenged with LPS (0.83mg/kg, i.p.; named LPS mice in following) or saline followed by weighing at 24 h i.p. and submitted to assessment of depressive-like behavior before euthanization for hypothalamus collection. Eighteen mice (*n* = 9/group), submitted to the hypothalamus proteomics, were selected for statistical analysis of behavioral changes in mice. The behavioral performance of LPS-related depression, including BW and food intake, SPT, TST, and FST (Supplementary Figure S2), was consistent with our previous publications (Wu et al., 2016, 2017). Briefly, LPS mice underwent more BW loss than the CON group due to less food intake. The reduction in sucrose preference indicates that LPS mice showed depression-related anhedonia. Moreover, LPS mice showed a significant increase in immobility compared with the control group in both the TST and FST. An elevated immobility time and anhedonia in the LPS group indicated that our depression mouse model was established. Additionally, to detect the local inflammation, proinflammatory cytokine expression in the hypothalamus was evaluated by qRT-PCR. The mRNA levels of IL-1β, TNF-α, and IL-6 in the LPS-depressed group were significantly higher than those of CON mice (*P* < 0.05, Figure 1).

### iTRAQ-Based Proteomics Analysis of the Hypothalamus

**FIGURE 2:**
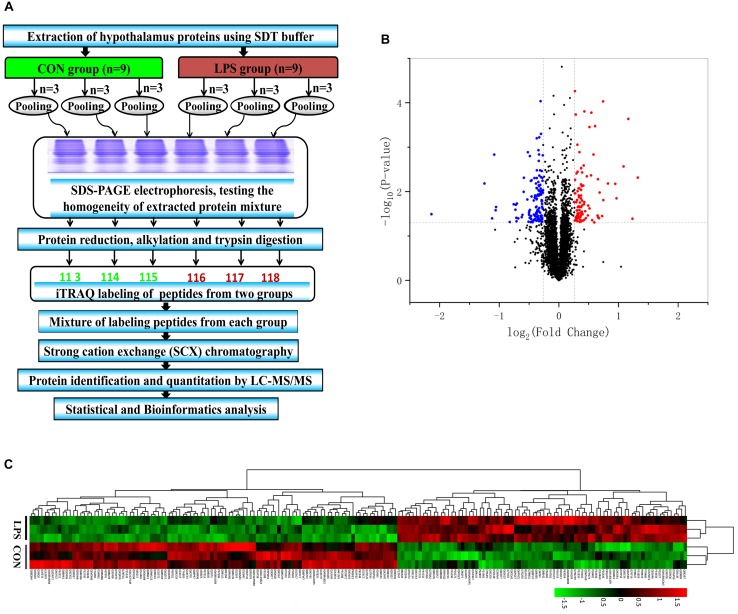
iTRAQ-based proteomic analysis of hypothalamus samples from LPS mice and controls. **(A)** Workflow diagram. The proteins were subjected to iTRAQ labeling followed by mass spectrometric analysis. **(B)** Volcano plot of all 4787 identified proteins quantified by proteomic analysis from the LPS and CON groups, respectively. One hundred eighty-seven proteins with at least 1.20-fold changes at *P* < 0.05 relative to the controls were identified in the two groups. Non-significantly changed, significantly downregulated, and upregulated proteins are shown in black, blue, and red, respectively. **(C)** Heatmap of the 187 different proteins in the comparing the LPS and CON groups. The heatmap was plotted based on the levels of the differential proteins. The heatmap data was normalized using *Z-scores*. Distinct separation was observed between control and LPS-depressed mice. Rows: Samples; Columns: proteins. The color intensity indicates the protein expressional level as displayed: red, highest; green, lowest.

After SCX fractionation, desalination and subsequent LC–MS/MS analysis (Figure 2A), 58,889 MS/MS spectra were acquired, of which 28,850 peptide spectra matched with the information in the database. In total, 23,252 unique peptides were identified. Additionally, 4,787 proteins were confirmed with at least one unique peptide and a 1% FDR. According to the criteria of *P-*value < 0.05, a unique peptide > 1 and fold changes > 1.20 or <0.83, 187 differential proteins ultimately exhibited significantly different expression between the groups (Supplementary Table S2). Among them, 83 proteins were upregulated and 104 were downregulated in the LPS group compared with that in the CON group (Figure 2B). The differential proteins in both groups were evaluated by hierarchical clustering analysis (Figure 2C).

**FIGURE 3:**
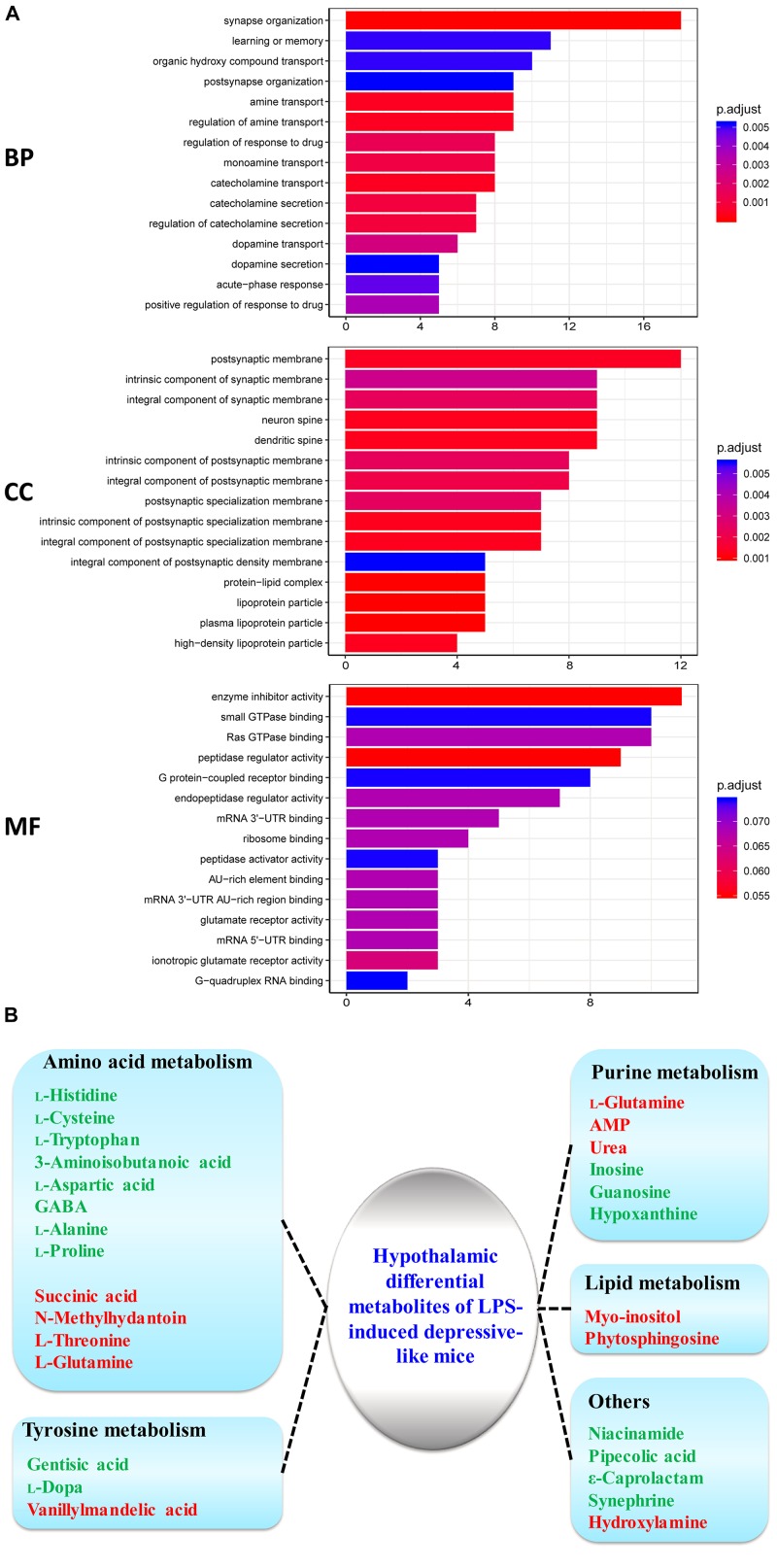
GO analysis of the identified proteins and metabolism process cluster of differential metabolites in the hypothalamus. **(A)** Gene Ontology (GO) classification of the identified differential proteins by DAVID annotation database. The results are summarized under three main GO categories: biological process (BP), cellular component (CC), and molecular function (MF). The *Y*-axis represents the GO terms that are associated with the protein set while *X*-axis represents the number of proteins. The color indicated the statistical differences of the GO terms via adjust *P*-value using Benjamini–Hochberg method. **(B)** Metabolism process of key differential metabolites detected by GC–MS for LPS/CON. Colored nodes represent input metabolites. Green: downregulated metabolites; Red: upregulated metabolites.

To obtain insight into the intracellular distribution and biological implication of the proteins with altered abundance, 187 differentially expressed proteins were analyzed by functional enrichment based on the Gene Ontology (GO), DAVID, and UniProt databases. Based on their functional features, 187 differential proteins were clustered into the categories related to BP, CC, and MF. The top 15 enriched terms were depicted in Figure 3A. Many differentially expressed proteins in the BP category were associated with synaptic function, transport and secretion of neurotransmitters, and inflammatory response. CC category analysis disclosed that the proteins were significantly clustered into synaptic membrane, neuron spine, and dendritic spine. Most identified proteins in MF analysis were concerned with peptidase regulator activity, glutamate receptor activity and nucleotide binding. Based on the KEGG and Reactome databases, the hypothalamic differential proteins were used for the pathway enrichment analysis. The Reactome and KEGG pathway analysis indicated EPHB-mediated forward signaling and neurological diseases-related pathways in the hypothalamus of LPS-induced depressed mice, respectively (Supplementary Figure S3).

### Integrated Analysis of Proteomics and Metabolomics

Our previous study had shown hypothalamic metabolite changes in the depressed mouse model through a GC-MS approach (Wu et al., 2017). Twenty-seven metabolites (10 high and 17 low expression) showed significantly different expression in the hypothalamus of LPS mice relative to controls (Figure 3B). Twelve metabolites (histidine, cysteine, tryptophan, 3-aminoisobutanoic acid, aspartic acid, GABA, alanine, proline, succinic acid, *N*-methylhydantoin, threonine, glutamine) were primarily implicated in amino acid metabolism (Figure 3B). Previous evidence has suggested amino acid metabolism in the brain mediates to neurotransmission, including glutamatergic transmission (Choudary et al., 2005; Masneuf et al., 2014; Wu et al., 2017).

Integrated analysis of bi-omics contributed to the exploration of neurological disorders, investigation of biological functions, and deciphering of canonical pathways and network relationships of differential expression molecules. The IPA database was selected to comprehensively analyze the metabolomic and proteomic data from the hypothalamus. In the biological function analysis of IPA based on data of proteomics and bi-omics, top five terms were showed in three categories (“Physiological system development and functions,” “Molecular and cell functions,” and “Disease and disorder”). We found that the ranked first term was same in both proteomics and bi-omics, as Behavior, Cell morphology and Neurological disease in three categories, respectively (Supplementary Figure S4). However, the term order ranked from the second to the fifth is different in the “Physiological system development and functions” and “Molecular and cell functions” categories. For example, “nervous system development and function” term ranked the third in proteomics data, while it ranked the second in the bi-omics. Additionally, some key terms such as Cellular function and maintenance, Cellular growth and proliferation, and Cell death and survival, were listed in both mono- and bi-omics analysis of “Molecular and cell functions” category, which indicated neurogenesis in depression. Moreover, in “Physiological system development and functions,” differentially expressed molecules were significantly enriched in the identified concepts, such as Behavior, and Nervous system development and function, which are strongly associated with mental diseases.

**FIGURE 4:**
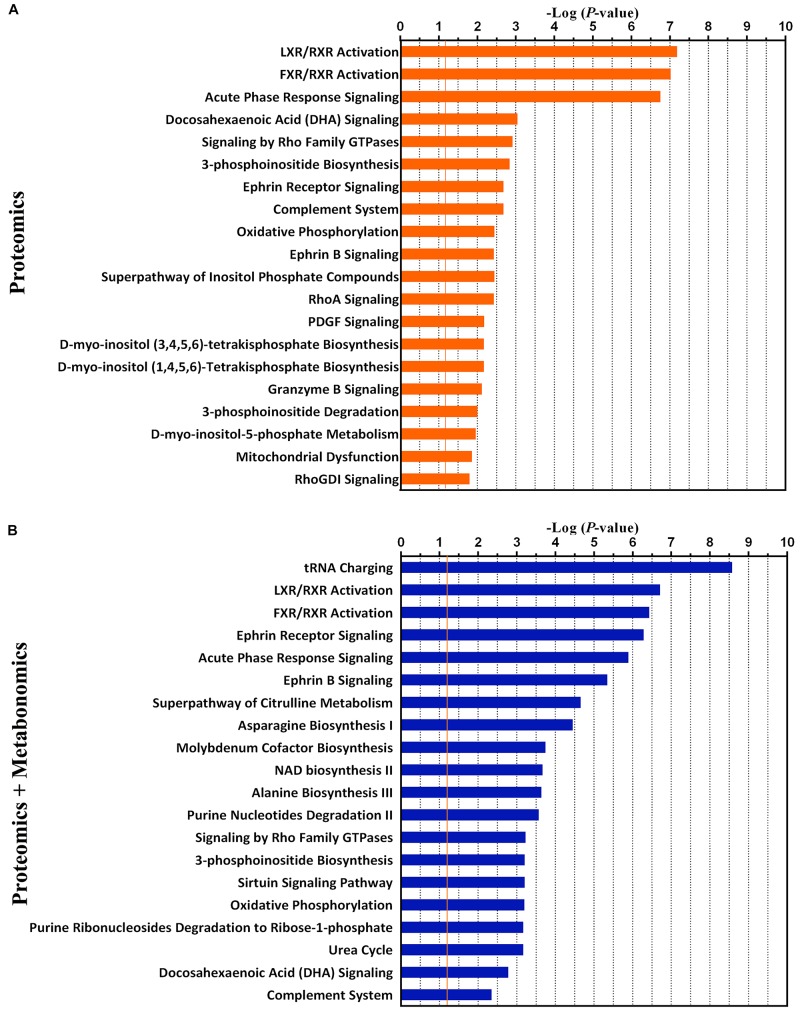
The top 20 canonical pathways of proteomics data only **(A)** and the combination of proteomics and metabolomics **(B)** by the Ingenuity Pathway Analysis (IPA). The *P*-value was obtained by Fisher’s exact test. The *Y*-axis represents the canonical pathway terms, and the *X*-axis represents the -Log_10_ (*P*-value).

**FIGURE 5:**
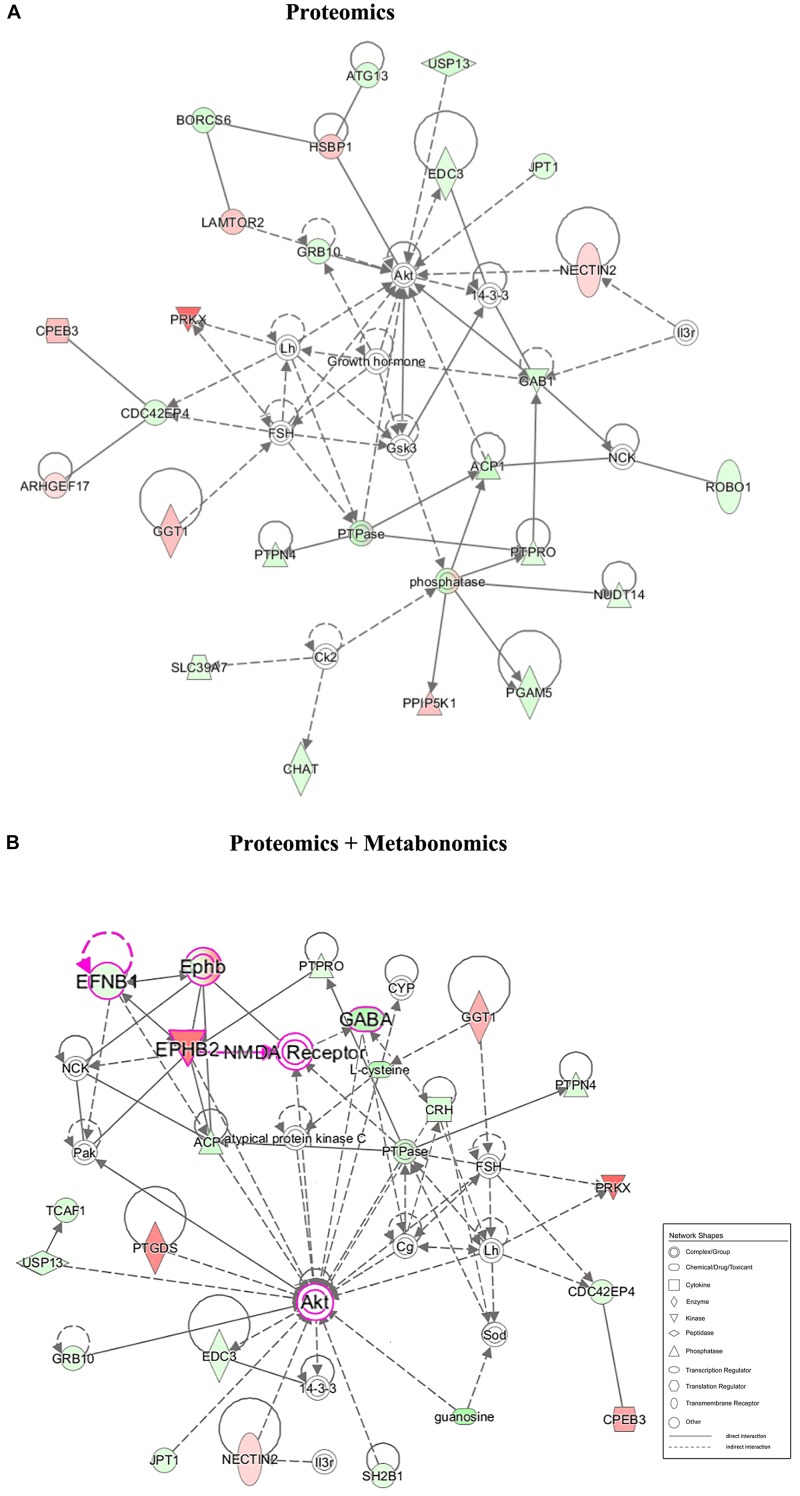
Correlation network between the significantly changed metabolites and proteins of the hypothalamus in the LPS group compared with that in the CON group. **(A)** Network-based proteomics data. **(B)** Network-based bi-omics (proteomics + metabolomics) data. Red symbols indicate upregulation, while green indicate downregulation, in the LPS group compared with that in the CON group. The molecular interaction network was generated based on the Ingenuity Pathway Knowledge Base. The hypergeometric distribution of the network was calculated using the right-tailed Fisher’s exact test. The gray solid lines and dotted lines show direct and indirect interactions or regulations of the two groups, respectively. Nodes of different shapes represent different meanings, similar to “Network Shapes.” Pink symbols in network B indicate a possible EPHB2-NMDAR-AKT cascade in the underlying pathogenesis.

Furthermore, the top 20 statistically canonical pathways of hypothalamic proteomics data and bi-omics data (proteomics and metabolomics) were summarized using the IPA (Supplementary Table S3 and Figure 4). The canonical pathways of hypothalamic differentially expressed proteins were showed in Figure 4A. The top five canonical pathways were liver X receptor (LXR)/retinoid X receptor (RXR) activation, farnesoid X receptor (FXR)/RXR activation, and acute phase response signaling, docosahexaenoic acid (DHA) signaling, and signaling by Rho family GTPases. In contrast, based on the combined dataset, the top five canonical pathways were tRNA charging, LXR/RXR activation, FXR/RXR activation, Ephrin receptor signaling, and acute phase response signaling (Figure 4B). Furthermore, as the top-ranking canonical pathway, the tRNA charging signaling involving ten dysregulated molecules (nine identified metabolites, 3 up- and 6 downregulated), mediated glutamatergic neurotransmission (Supplementary Figure S5). It is also related to the imbalance of amino acid metabolism and protein breakdown-biosynthesis catabolism (Wolfe, 2002). By comparing the pathway analysis between the mono- and bi-omics, there were pathways shared in both (such as LXR/RXR activation, FXR/RXR activation, Ephrin receptor signaling, acute phase response signaling, and complement system). These pathways involved in key proteins or metabolites play essential roles in regulating neurotransmitter imbalance, neural plasticity, and neuroinflammation and neuroimmunity of the hypothalamus. LXR/RXR activation, which is involved in the regulation of the inflammatory response and lipid metabolism. Additionally, the acute phase response signaling, often presented in depressed patients (Wang et al., 2018), was also shown in the present LPS depression model. The most important is Ephrin receptor signaling, as the fourth top-ranked statistically canonical pathways in the bi-omics, which regulates neuroinflammation and nervous system development through mediating glutamatergic neurotransmission, and neuroplasticity (Dines and Lamprecht, 2016; Wan et al., 2018). Given several key proteins within the pathway, such as Ephrin-B1 (EFNB1) and EPHB2, Ephrin receptor signaling may participate in inflammation-associated depression. The proprietary IPA knowledgebase was used to construct the causal networks for either the significantly changed proteins or proteins integrated metabolites of the hypothalamus (Figure 5). The data showed that the core node was AKT signaling for both networks. And two networks shared lots of differential expression proteins and some molecule complexes. However, with the contribution of the metabolomics analysis, it was interesting that the data suggested that Ephrin receptor signaling, the glutamatergic pathway, and AKT signaling were critical pathway in the depression (Figure 5B), which were selected for further research.

**FIGURE 6:**
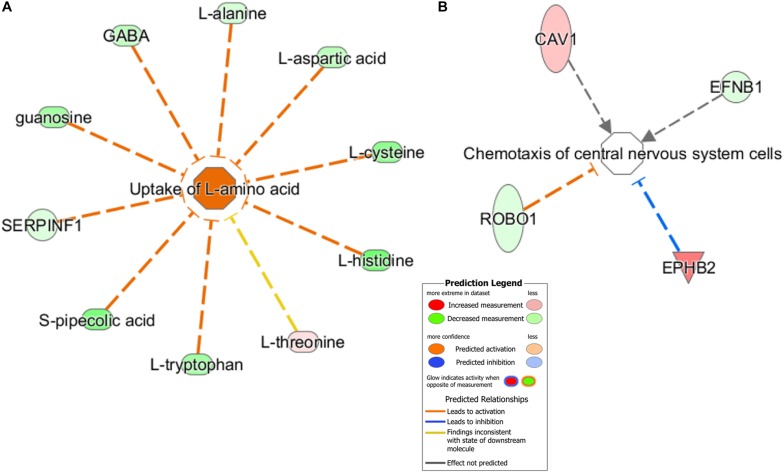
Predicted biological functions of the significantly changed metabolites and proteins were identified by IPA. **(A)** “Uptake of L-amino acid” and **(B)** “Chemotaxis of central nervous system cells.” Each molecule node is connected to the relevant functional nodes. Molecule symbols in red were upregulated while green symbols were downregulated in the LPS group. Different colored lines and nodes represent different meanings, similar to “Prediction Legend.”

Furthermore, using the predictive function analysis, these differential metabolites and proteins were significantly involved in the “uptake of L-amino acid” (Figure 6A) and “chemotaxis of CNS cells” (Figure 6B). The “Uptake of L-amino acid”-related differential metabolites and some differential proteins, including key receptors and catalytic enzymes, were mainly involved in the glutamatergic pathway (Supplementary Figure S5), which is supported by our previous hypothalamic metabolic profiling of LPS-depressed mice (Wu et al., 2017). The predicted “Chemotaxis of CNS cells” function contained functional proteins, such as EPHB2 and EFNB1, which are implicated in the regulation of glutamatergic receptors and synaptic plasticity.

### Validation of Key Protein Expression in the Glutamatergic Pathway and Ephrin Receptor Signaling

**FIGURE 7:**
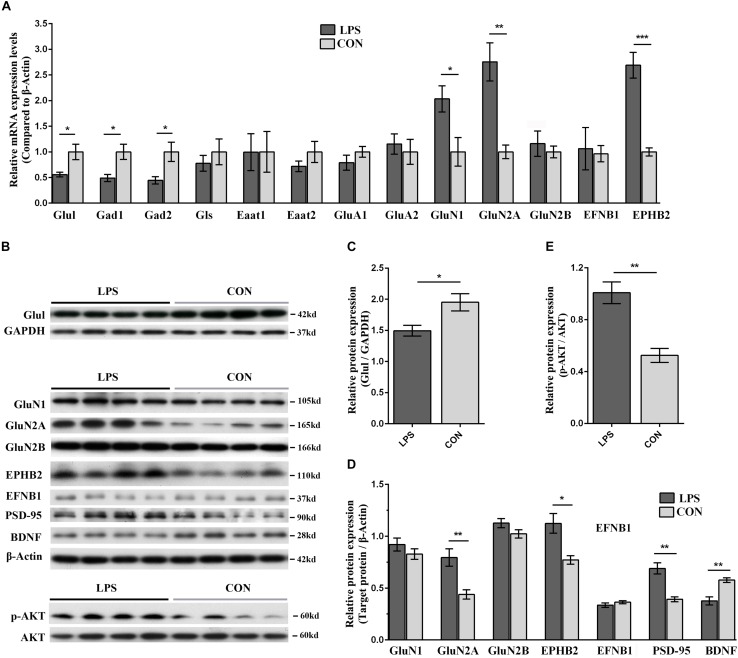
Validation of key players in Ephrin receptor signaling and glutamatergic transmission in the hypothalamus of LPS-induced depressed mice in both the mRNA and protein levels. **(A)** Transcriptional profiling of glutamatergic transmission-related proteins and Ephrin receptor signaling-related proteins. The mRNA changes were detected by qRT-PCR. All samples were normalized to the β-Actin gene as a control. The fold increase or decrease was relative to the CON group as the reference. **(B)** Immunoblotting validation of centering on EPHB2-NMDAR-AKT cascade related key proteins. The abundance of Glul, GluN1, GluN2A, GluN2B, EPHB2, EFNB1, PSD-95, BDNF, p-AKT, and AKT was determined using their corresponding specific antibodies. Equal amounts of protein fractions were loaded. **(C–E)** The expression of Glul **(C)**, GluN1, GluN2A, GluN2B, EPHB2, EFNB1, PSD-95, BDNF **(D)**, p-AKT, and AKT **(E)** was quantified according to the corresponding bands of proteins in **(B)**. GAPDH and β-Actin protein were used as the internal reference marker and loading control for normalization, respectively. All samples were validated in triplicate. The bands for the same proteins were analyzed by densitometry using Quality One software (version 4.6.6). Student’s *t*-test was utilized to analyze the significant difference between two groups. Values are presented as the means ± SEM (*n* = 5/group in qRT-PCR test, *n* = 4/group in WB test). ^∗^*P* < 0.05, ^∗∗^*P* < 0.01, ^∗∗∗^*P* < 0.001, LPS vs. CON.

Based on the aforementioned results of pathway analysis and network analysis, we hypothesized that hypothalamic neuroplasticity in inflammation-associated depression might be regulated by Ephrin receptor signaling via influencing the glutamatergic transmission and AKT signaling cascade. To test this hypothesis, qRT-PCR and WB were applied to validate the expression of key genes and proteins in Ephrin receptor signaling, and the glutamatergic pathway in the hypothalamus. Next, changes in the levels and phosphorylation status of AKT in the hypothalamus were analyzed after LPS intervention. EFNB1 and EPHB2, which were raised and lowered in the present iTRAQ proteome results, respectively, belonged to core molecules of the Ephrin receptor signaling pathway. The mRNA level of EFNB1 and EPHB2 in LPS mice showed no significant difference (*P* = 0.105) and a significant increase (*P* < 0.001) compared with the CON group, respectively (Figure 7A). The protein expression levels of EPHB2 and EFNB1 were verified by WB. EPHB2 was significantly elevated in the LPS group (*P* < 0.05). Nevertheless, EFNB1 showed no significant alteration compared with the CON group (*P* = 0.316, Figures 7B,D).

Regarding the glutamatergic pathway in the hypothalamus, the specificity of molecular alterations, such as glutamatergic enzymes, transporters and receptors, urgently required further verification in an inflammation-associated depression model. The mRNA expression levels of key enzymes including glutamine synthetase (Glul), glutaminase (Gls), glutamate decarboxylase (Gad) 1, Gad2, those of glutamate transporters such as excitatory amino acid transporter (Eaat) 1, Eaat2, and those of glutamatergic receptors including glutamate receptor ionotropic-AMPA (α-amino-3-hydroxy-5-methylisoxazole-4-propionic acid) type subunit 1 (GluA1), GluA2, glutamate receptor ionotropic-*N*-methyl-D-aspartate 1 (NMDAR1, GluN1), NMDAR2A and 2B (GluN2A and 2B), were determined by qRT-PCR. The mRNA levels of Glul, Gad1, and Gad2 in LPS-depressed mice showed significant reduction compared with those in the controls (*P* < 0.05), and GluN1 and GluN2A were significantly elevated in LPS mice (GluN1, *P* < 0.05; GluN2A, *P* < 0.01; Figure 7A). The protein levels of Glul, GluN1, and GluN2A & 2B were quantified by WB. Glul and GluN2A in LPS-depressed mice were significantly decreased and increased compared with that in the CON group, respectively (Glul, *P* < 0.05, Figures 7B,C; GluN2A, *P* < 0.01, Figures 7B,D). These results indicated that the GluN2A subunit was critical in neuroinflammation-related depression.

The disturbance of glutamatergic transmission and its related dysfunction of synaptic plasticity have been speculated as the underlying mechanisms of depression (Ferreira et al., 2015; Francija et al., 2018). Our integrated network analysis also predicted that the EPHB2 and EFNB1 interactions play an important role in regulating NMDARs (Figure 5). Although studies emphasized the role of the GluN2A and AKT signaling cascade in LPS-induced depression (Francija et al., 2018; Li et al., 2018), it remains open how Ephrin receptor signaling acts on NMDARs and AKT signaling contributes to decreased neuroplasticity and aberrant behavior upon LPS treatment. To probe into possible mechanisms, we also selected an additional three proteins involved in this biological process (PSD-95, p-AKT, and BDNF) for immunoblotting. PSD-95, a major postsynaptic scaffolding protein, directly binds to GluN2A and GluN2B and participates in the trafficking, anchoring, and internalization of NMDARs (Kornau et al., 1995; El-Husseini et al., 2000). BDNF served as an indicator of neuroplasticity damage in the hypothalamus. NMDAR-associated protein PSD-95 was significantly increased and BDNF was significantly reduced in the LPS group (*P* < 0.01, Figures 7C,D). Furthermore, as network analysis, AKT was predicted as the most significantly regulated molecule of the entire inter-relationship (Figure 5). Our results revealed increased p-AKT in the LPS group (*P* < 0.01, Figures 7B,E).

## Discussion

One of the molecular mechanisms of depressive disorders is associated with neuroinflammation (Dantzer et al., 2008; Savitz et al., 2013; Wu et al., 2017; Francija et al., 2018). To meticulously explore how inflammation modulates depressive symptoms, the establishment of an inflammation-associated depression model is necessary. The LPS-induced depression animal model mimics some depressive features of patients with chronic inflammation-associated diseases, such as systemic infections, cancers and autoimmune diseases (O’Connor et al., 2009; Walker et al., 2013; Wu et al., 2016; Francija et al., 2018). To the best of our knowledge, this is the first study integrating proteomics and metabolomics to systematically investigate the molecular mechanisms of inflammation-associated depression in the hypothalamus. Hypothalamic metabolome and proteome changes during inflammatory disequilibrium were mainly enriched into the perturbations of Ephrin receptor signaling, glutamatergic transmission and inflammation related signaling. The communications among these pathways may contribute to the impaired synaptic plasticity of the hypothalamus and depression-like behavior. Our results were attributed to the following significant findings: (1) LXR/RXR activation, FXR/RXR activation, and acute phase response signaling were primarily clustered into immunity and inflammation-related signaling, which indicated initiating factor of LPS-induced depression mouse model: “neuroinflammation.” (2) Perturbation of glutamatergic transmission and Ephrin receptor signaling were significant among the changed pathways; (3) The impairments of hypothalamic neuroplasticity were regulated by Ephrin receptor signaling, which influenced the GluN2A mediated glutamatergic transmission and AKT signaling; (4) EPHB2 and GluN2A may be potential therapeutic targets for depression.

Using iTRAQ-based proteomics approach, we identified 187 differentially expressed proteins in the hypothalamus of LPS-induced depressed group compared to controls. Among these, most proteins in BP category analysis were significantly clustered into transport related-terms, such as amine, ammonium, catecholamine, monoamine and dopamine transport, which might indicate the overrepresentation of “amino acid transport” function. Additionally, synaptic function and inflammatory response were also enriched keys for some proteins in the BP analysis. The disequilibrium of these biological processes might underlie the observed behavioral differences. According to the IPA-based pathway and network analysis, without doubt, bi-omics provided more evidences than single omics. The bi-omics network revealed interactions between proteins and metabolites, and provided visualization for interactome among Ephrin receptor signaling, glutamatergic transmission and AKT signaling in the hypothalamus. Twenty-seven differential metabolites in metabolomics analysis, even far less than the number of identified differentially expressed proteins, provided critical information in exploration of key pathways and molecular mechanisms in depression. Integrated analysis of multiple-omics data is conducive to better understanding of potential molecular mechanisms in depression (Misra et al., 2018; Schubert et al., 2018).

In the CNS, some enzymes, transporters, and receptors involved in glutamatergic transmission are associated with the mechanisms of depression (Walker et al., 2013; Masneuf et al., 2014; Zhou et al., 2018). Gad1, Gad2 and Glul, are the key regulatory enzymes that maintain the metabolic homeostasis of GABA and glutamate in the glutamate-GABA-glutamine cycle between neurons and astrocytes (Veeraiah et al., 2014; Wang et al., 2016). With the catalysis of Gad1 and Gad2, the neuronal glutamate is transformed into GABA. Glul, one of the astrocyte-specific enzymes, catalyzed the conversion of glutamate into glutamine, and its levels were decreased in the cortical areas of MDD patients (Choudary et al., 2005). Herein, the levels of Gad1, Gad2 and Glul were reduced in the hypothalamus of LPS mice. Our previous study showed a significant augmented level of glutamine, an obvious decrease in GABA and no difference in glutamate production in the hypothalamus of LPS-depressed mice (Wu et al., 2017). This may be indicative of the fact that Gad1, Gad2, and Glul were implicated in the LPS-induced dysfunction of glutamate-GABA-glutamine neurotransmitter cycling. Additionally, as critical excitatory glutamate transporters in glutamatergic metabolism, Eaat1 and Eaat2 are the most abundant Na+-dependent glutamate carriers expressed predominantly in brain gliocytes (Parkin et al., 2018). The primary functions of Eaat1 and Eaat2 are to modulate glutamatergic excitability and prevent the spilling over of glutamate beyond the synapse (Parkin et al., 2018). Considerable evidence has exhibited a significant downregulation of Eaat1 and Eaat2 in the bilateral cortical areas of depressed individuals (Choudary et al., 2005). However, our results showed unaltered Eaat1 and Eaat2 in the hypothalamus of the LPS mice. Eaat1 and Eaat2 may not be key alterations in the hypothalamus of the LPS-induced depression model. Overall, the above key regulatory enzymes of glutamatergic transmission were involved in the dynamic equilibrium of the glutamate-GABA-glutamine neurotransmission and LPS-induced depressive-like behavior (Choudary et al., 2005; Veeraiah et al., 2014; Yin et al., 2016).

Regarding glutamatergic receptors, recent reports have demonstrated that alterations of NMDAR subunits mediate synaptic plasticity impairment in the PFC and hippocampus of LPS-induced depression mice (Walker et al., 2013; Francija et al., 2018). NMDARs, comprising GluN1, GluN2A and 2B, and GluN3 subunits, have particular features, such as the detectors of coincident presynaptic and postsynaptic activities (Ferreira et al., 2015). The hypothalamus mRNA level of GluN1 in LPS mice was significantly increased even with no significant change in its protein expression. This may be due to transcriptional regulation of protein expression. Furthermore, the mRNA and protein levels of GluN2A were all significantly increased in the hypothalamus of LPS mice. Consistent with our findings, Francija et al. (2018) suggested that GluN2A absence abolished LPS-induced depression. Taken together, our results supported that the GluN2A subunit was critical in neuroinflammation-related depression.

For the first time, Ephrin receptor signaling was found in the hypothalamus of the LPS-induced depression model. As the fourth-ranking canonical pathway, Ephrin receptor signaling mediates glutamatergic neurotransmission and neuroplasticity in injury and inflammation of the CNS (Coulthard et al., 2012; Wan et al., 2018). The Eph tyrosine kinase receptors were subdivided into 9 EPHA receptors (EPHA1-8 and EPHA10) and 5 EPHB receptors (EPHB1-4 and EPHB6), based on their binding to two subclasses of cognate ligands, Ephrin-As and Ephrin-Bs (Dines and Lamprecht, 2016; Taylor et al., 2017). The EPHB/Ephrin-Bs, which are distributed extensively and provide unique bidirectional signaling between neurons and astrocytes, have attracted considerable attention because of their regulation of neuronal maturation and synaptic plasticity (Koeppen et al., 2018). Dysfunction of EPHB2 and its ligand EFNB1 has been implicated in brain disorders, such as cognition disorders and depression (Zhang et al., 2016; Koeppen et al., 2018). Even in other subtypes of depression animal models, EPHB2 was preliminarily found to regulate neuronal development, NMDAR function, and synaptic plasticity of the hippocampus and PFC (Zhang et al., 2016; Zhen et al., 2018). Little has been reported on the function of EPHB2 and its ligand EFNB1 in the hypothalamus of inflammation-related depression. Our hypothalamic proteomic results indicated elevated EPHB2 and reduced EFNB1 in LPS-depressed mice. Our WB and qPCR detection further confirmed that EPHB2 mRNA and protein expression levels were significantly increased in the hypothalamus of LPS-depressed mice. However, the EFNB1 mRNA and protein levels failed to show any significant difference, as validated in the qRT-PCR and WB assays, indicating unaltered levels of EFNB1. Our results explicitly delineated that EPHB2 is the most significant altered molecule of Ephrin receptor signaling in the hypothalamus of the LPS-induced depressive phenotype. EPHB2 is not only involved in glutamatergic neurotransmission but also simultaneously participates in the inflammation regulation process in some brain diseases including stroke, anxiety and Alzheimer’s disease (Coulthard et al., 2012; Dines and Lamprecht, 2016; Ernst et al., 2019). Cumulatively, our present study and aforementioned studies showed that EPHB2 is intimately involved in inflammation-related depression.

Next, our integrated network analysis implied that EPHB2 might participate in the regulation of NMDARs, particularly GluN2A subunit. EPHB2, a transmembrane receptor, has an extracellular N-terminal ligand-binding domain and an intracellular Carboxy-terminal PDZ (postsynaptic density protein-95, disk large, zona occludens-1)-binding domain (Kayser et al., 2006; Taylor et al., 2017). A direct interaction between EPHB2 and NMDARs via the PDZ-binding domain (Dalva et al., 2000) resulted in NMDAR subunit GluN2B clustering and enhanced NMDAR-dependent Ca2+ flux (Takasu et al., 2002). EPHB2-null mice displayed a reduction in NMDARs and abnormal NMDAR-dependent synaptic plasticity (Henderson et al., 2001). Additionally, as a neuronal PDZ protein, PSD-95 can specifically and directly interact with AMPAR or NMDAR subunits through PDZ domain (Kornau et al., 1995; El-Husseini et al., 2000; Henderson et al., 2001; Zhang et al., 2016). These studies suggest that EPHB2 plays a crucial role in the regulation of NMDARs via its PDZ domain. Zhang et al. (2016) and Hanamura et al. (2017) reported that EPHB2 modulates the expression and synaptic localization of AMPAR subunit GluA1 and 2 or the NMDAR subunit GluN2B through interactions with the PSD-95-binding domain PDZ. Our results revealed that EPHB2, PSD-95, and GluN2A expression increased in the hypothalamus of depressed mice. Thus, we considered the possibility that hypothalamic-upregulated EPHB2 might facilitate the cluster of GluN2A via PDZ interaction with PSD-95 in the development of depression.

NMDARs activation has distinct consequences on synaptic plasticity (Williams and Schatzberg, 2016; Francija et al., 2018). It was demonstrated that NMDARs regulated several downstream signal transduction pathways correlated to the synaptic plasticity of depression (e.g., cAMP-PKA, RAS-ERK, MAPK, and PI3K-AKT) (Yoshii and Constantine-Paton, 2010; Marsden, 2013; Leal et al., 2014). Here, our integrated network analysis also emphasized that the AKT signaling cascade plays a potential role in the synaptic plasticity of LPS-induced depression. Increased GluN2A was accompanied by a significant increase in p-AKT and a reduction of the neuroplasticity indicator BDNF, which exhibits abnormal synaptic plasticity in the hypothalamus of LPS mice. Taken together, EPHB2 overexpression, which is connected to PSD-95, facilitates the recruitment of GluN2A, which further acts on the AKT signaling. AKT phosphorylation engages in the impairment of hypothalamic synapse plasticity, which is ultimately attributed to depressive-like behavior upon an inflammation challenge (Francija et al., 2018).

Bidirectional EPHB/Ephrin-Bs signaling is a major form of contact-dependent communication between brain cells (Dines and Lamprecht, 2016; Wan et al., 2018). Our present study mainly focuses on the forward signaling as the regulation of EPHB2 on postsynaptic GluN2A as well as its downstream via PSD-95. Studies have shown that EPHB2 reversely triggers presynaptic differentiation via its Ephrin-Bs binding domain (Kayser et al., 2006; Wan et al., 2018). Whether EPHB2/Ephrin-Bs reverse signaling affects the glutamate system balance needs to be further investigated in future studies.

**FIGURE 8:**
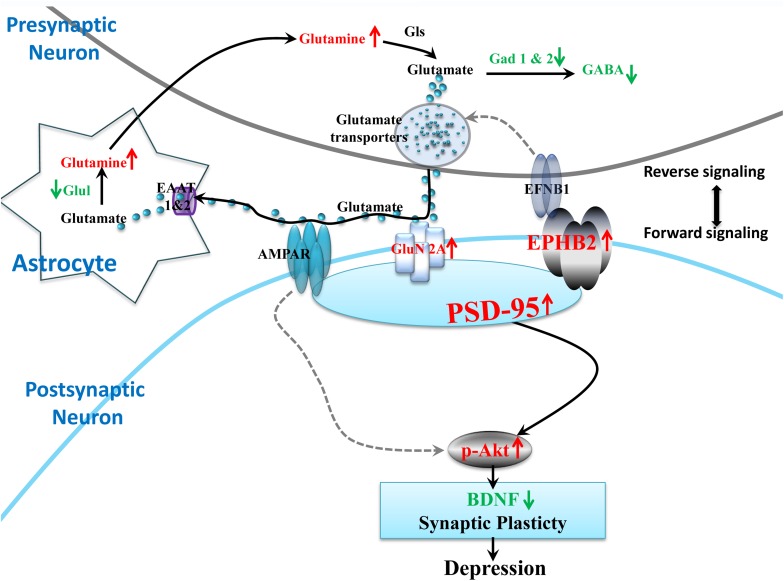
Proposed model of the EPHB2 signaling cascade that regulates the synaptic plasticity through the phosphorylation of AKT under neuroinflammation-associated perturbation of glutamatergic transmission. LPS intervention influences the glutamatergic system, including decreased important enzymes of the glutamate metabolic pathway (Glul, Gad1, Gad2), and the elevated pivotal GluN2A receptor of glutamatergic synapse. Additionally, LPS elevates the expression of EPHB2, PSD-95 and phosphorylation of AKT (p-AKT) in the hypothalamus. EPHB2 may regulate neuroinflammation stress vulnerability by modulating synaptic plasticity via possibly facilitating GluN2A traffic, and then activating their specifically interacted protein, PSD-95. Next, the phosphorylation of AKT leads to the impairment of synaptic plasticity and ultimately depressive-like behavior. Molecular symbols with the up red arrow indicate upregulation, while those with the green arrow indicate downregulation in the LPS group compared with that in the CON. The solid black arrow lines show glutamatergic transmission interactions or synaptic plasticity regulation. The gray dotted arrow lines show regulation cited previously.

Taken together, the bioinformatics and validated results in the hypothalamus showed that the perturbations of Ephrin receptor signaling and glutamatergic transmission and its communications may contribute to the impaired synaptic plasticity of the hypothalamus and depression-like behaviors. Based on our results, Ephrin receptor signaling in the hypothalamus is critical for regulating synaptic plasticity and the vulnerability to LPS treatment. We integrated glutamatergic transmission and Ephrin receptor signaling into a brief plot to propose a signaling cascade of EPHB2-GluN2A-AKT in regulating hypothalamic synapse plasticity (Figure 8). It is worth mentioning that some studies have found that EPHB2 also interacts with AMPARs via PSD-95 to modulate synaptic plasticity in the chronic social defeat stress depression subtype (Zhang et al., 2016), however, our study did not reveal alteration of AMPAR subunits GluA1 and GluA2 under neuroinflammation stress. However, it does not exclude the possibility that AMPARs play a role in regulating neuroplasticity through PSD-95 in inflammation-related depression (Zhang et al., 2017).

There are several limitations within this study. First, the integrated analysis cannot cover every aspect of the biological process, due to a much narrower metabolomic profile than proteomic changes. Second, since many low-abundant metabolites (e.g., lipids, neurotransmitters, steroids, and eicosanoids) are not detectable by GC-MS, the combination of GC-MS with other analytical tools (e.g., NMR, LC-MS or other more specific, targeted techniques) should be considered in future studies. Analogously, regarding the iTRAQ and label free approaches, two very popular quantitative proteomic analysis methods, each has its own advantages and can complement each other. Only the iTRAQ approach was employed here. The combination of the two methods should be able to cover more images. Third, we only confirmed as dysregulated a limited set of proteins that are involved in Ephrin receptor signaling and glutamatergic transmission by qRT-PCR and WB. Subsequent studies should focus on validating additional identified proteins from hypothalamic proteomics to better illuminate the physiopathologic mechanisms of depression. Fourth, although we preliminarily found perturbations in the expression of EPHB2 and GluN2A involved in Ephrin receptor signaling and glutamatergic transmission in the hypothalamus of LPS-induced depressed mice, the precise mechanisms of such perturbation require further investigation in *in vitro* and *in vivo* experiments.

## Conclusion

Proteomics integrated with metabolomics were implemented to investigate the changes in proteins and metabolites in the hypothalamus of LPS-induced depressed mice. The perturbation of glutamatergic transmission and Ephrin receptor signaling may contribute to the impaired synaptic plasticity of the hypothalamus and depression-like behavior. Understanding the role of EphB2 and GluN2A in depression provides novel molecular intervention targets for future treatment. This research gains new insight into the biochemical mechanisms of inflammation-associated depression.

## Data Availability Statement

The datasets generated for this study can be found in the ProteomeXchange, PXD016040, https://www.ebi.ac.uk/pride/archive/projects/PXD016040.

## Ethics Statement

The animal study was reviewed and approved by the Gansu Provincial Hospital Ethics Committee, Gansu Provincial Hospital. Written informed consent was obtained from the owners for the participation of their animals in this study.

## Author Contributions

XG and YW conceived and designed the experiment. YW, ZW, YoL, CW, YJ, and HX performed the experiment. PC, YuL, ZW, and XQ analyzed the data. JJ, RG, WW, and ZL contributed to the reagents, materials, and analysis tools. XG and YW wrote the manuscript and supervised the project. All authors accepted the final version of the manuscript.

## Conflict of Interest

The authors declare that the research was conducted in the absence of any commercial or financial relationships that could be construed as a potential conflict of interest.

## Footnotes

1 http://www.uniprot.org

2 https://david.ncifcrf.gov

3 https://yulab-smu.github.io/clusterProfiler-book/chapter12.html#bar-plot

4 http://www.ingenuity.com

5 https://reactome.org/

6 https://omictools.com/impala-tool

7 http://www.genome.jp/kegg/

8 http://www.pantherdb.org/
