# Supplementary material for: Perturbation of Ephrin Receptor Signaling and Glutamatergic Transmission in the Hypothalamus in Depression Using Proteomics Integrated With Metabolomics
Source: Front Neurosci. 2019 Dec 17;13:1359. doi: 10.3389/fnins.2019.01359 (PMC6928102; doi:10.3389/fnins.2019.01359)

**Figure S1. The experimental timeline and study design.** Abbreviations: LPS = lipopolysaccharide (LPS)-induced depressed group, CON = control group, BW = body weight, SPT = sucrose preference test, TST = tail suspension test, FST = forced swimming test, iTRAQ = isobaric tags for relative and absolute quantitation, GC-MS, gas chromatography–mass spectrometry.


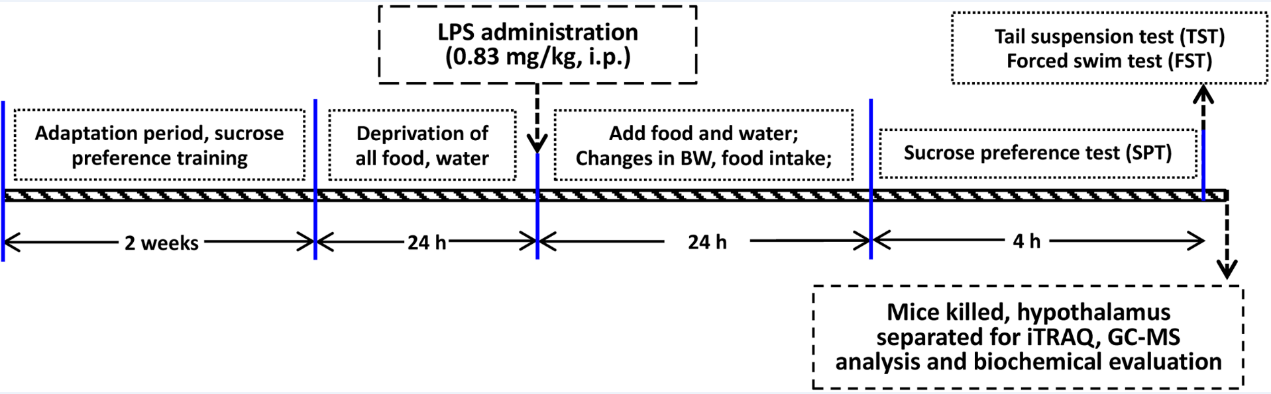


**Figure S2. Assessment of the lipopolysaccharide (LPS)-induced mouse model of depression.** Eighteen mice, submitted the hypothalamus proteomics, were selected for statistics of behavioral changes in mice. **(A)** Changes in body weight (BW) during the 24 h following LPS exposure. **(B)** Changes in food consumption during the 24 h following LPS exposure. **(C)** Sucrose preference in the sucrose preference test (SPT) 24 h after LPS treatment. **(D, E)** Immobility times in the tail suspension test (TST) and forced swimming test (FST), respectively. Student’s *t*-test was used to analyze the significant difference between two groups. Values are shown as means ± SEM (n = 9 / group). **P* < 0.05, ***P* < 0.01, ****P* < 0.001, LPS vs. CON. *Abbreviations:* LPS = LPS-induced depressed group, CON = control group, BW = body weight, SPT = sucrose preference test, TST = tail suspension test, FST = forced swimming test, SEM = standard error of the means.


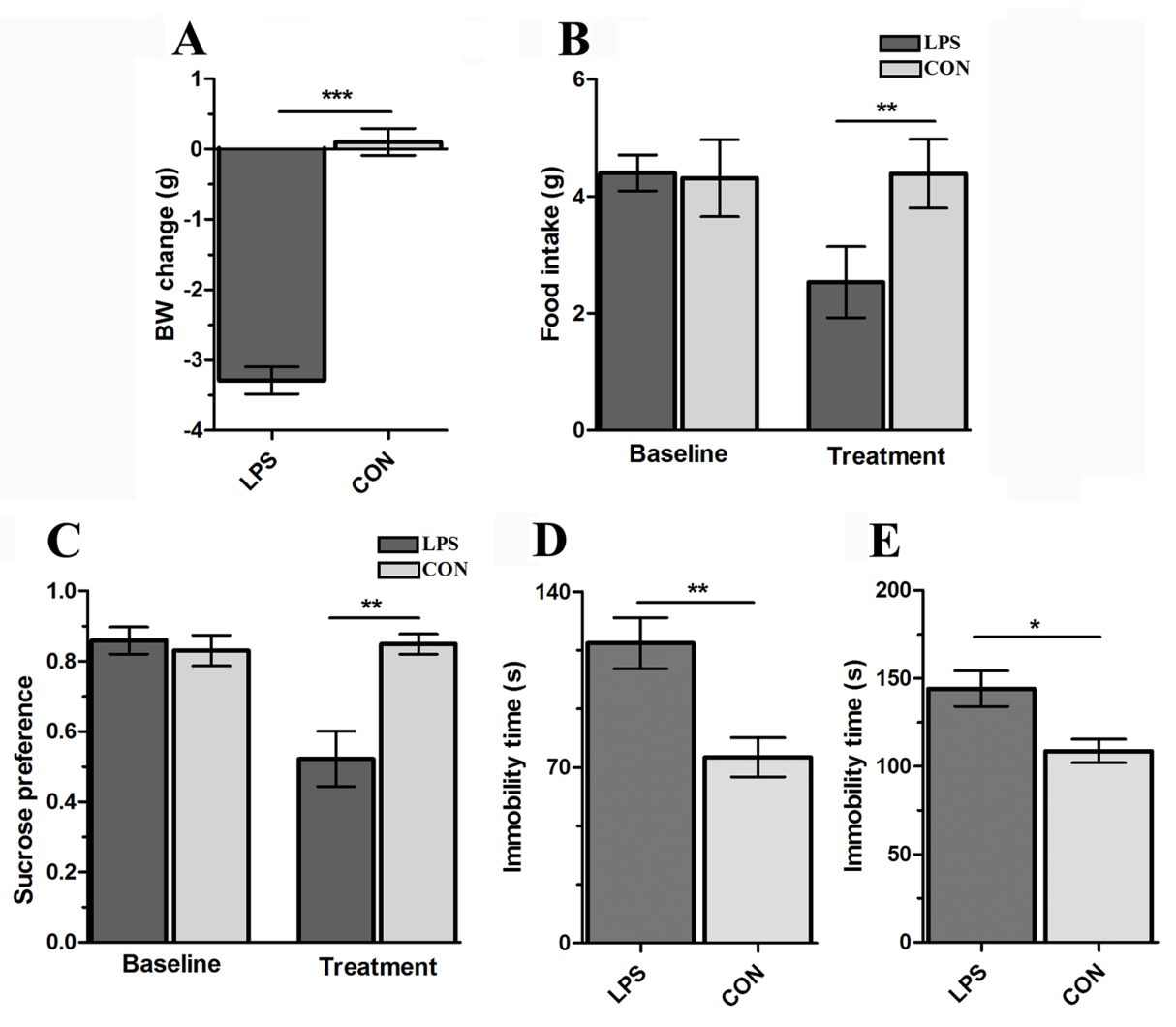


**Figure S3. Enrichment analysis of Gene Ontology (GO) pathway terms based on the KEGG and Reactome databases for the differentially expressed proteins in the hypothalamus.** The Y-axis represents the pathway terms that are associated with the protein set while X-axis represents the number of proteins. The color indicates the significant differences between the pathway terms via –Log _10_ (*P*-value).

**
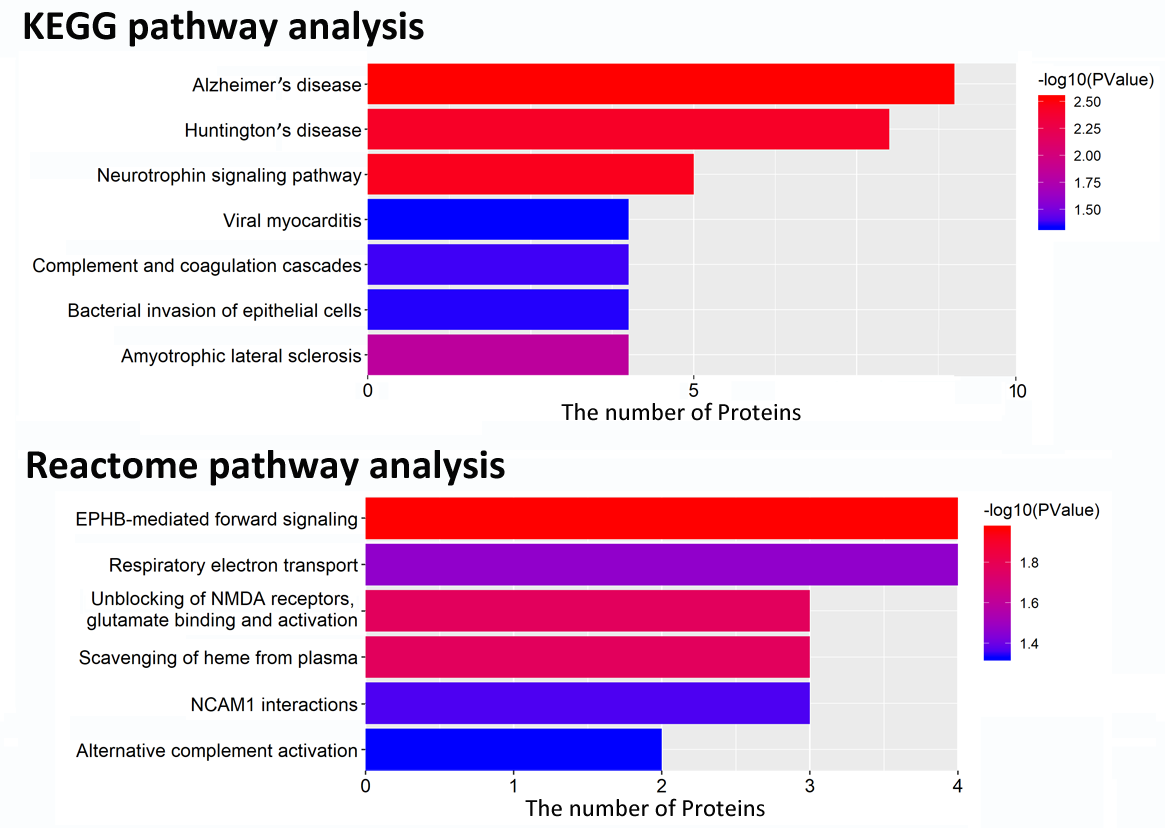
**

**Figure S4. Biological Function analysis in IPA for the hypothalamic proteomics data and proteomics combined metabolomics dataset**. Three functional categories, including Physiological system development and functions, Molecular and cell functions, Disease and disorder, were displayed. The number of differentially expressed molecules was indicated at the end of functional terms.


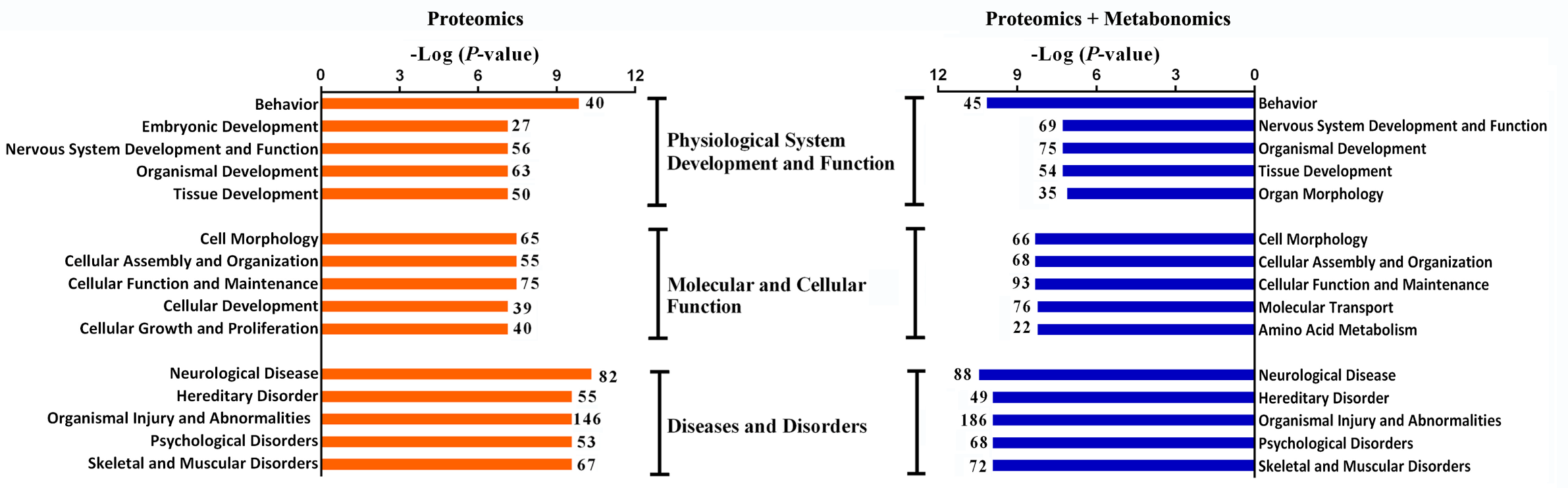


**Figure S5. An overview of the hypothalamic glutamatergic pathway covered key differential metabolites and proteins.** Oval nodes are metabolites, and rectangle nodes are proteins. Red-labeled molecules show increased level in the hypothalamus of LPS-induced depressive-like mice, and green-labeled molecules show decreased levels, grey-labeled molecules show no changed. TCA, tricarboxylic acid; GABA, gama-aminobutyric acid; Glul, Glutamine synthetase; Gls, Glutaminase; AMPAR, α-amino-3-hydroxy-5-methylisoxazole-4-propionic acid receptor; Gad1 & 2, Glutamate decarboxylase 1 & 2; NMDAR, N-methyl-D-aspartate receptor.


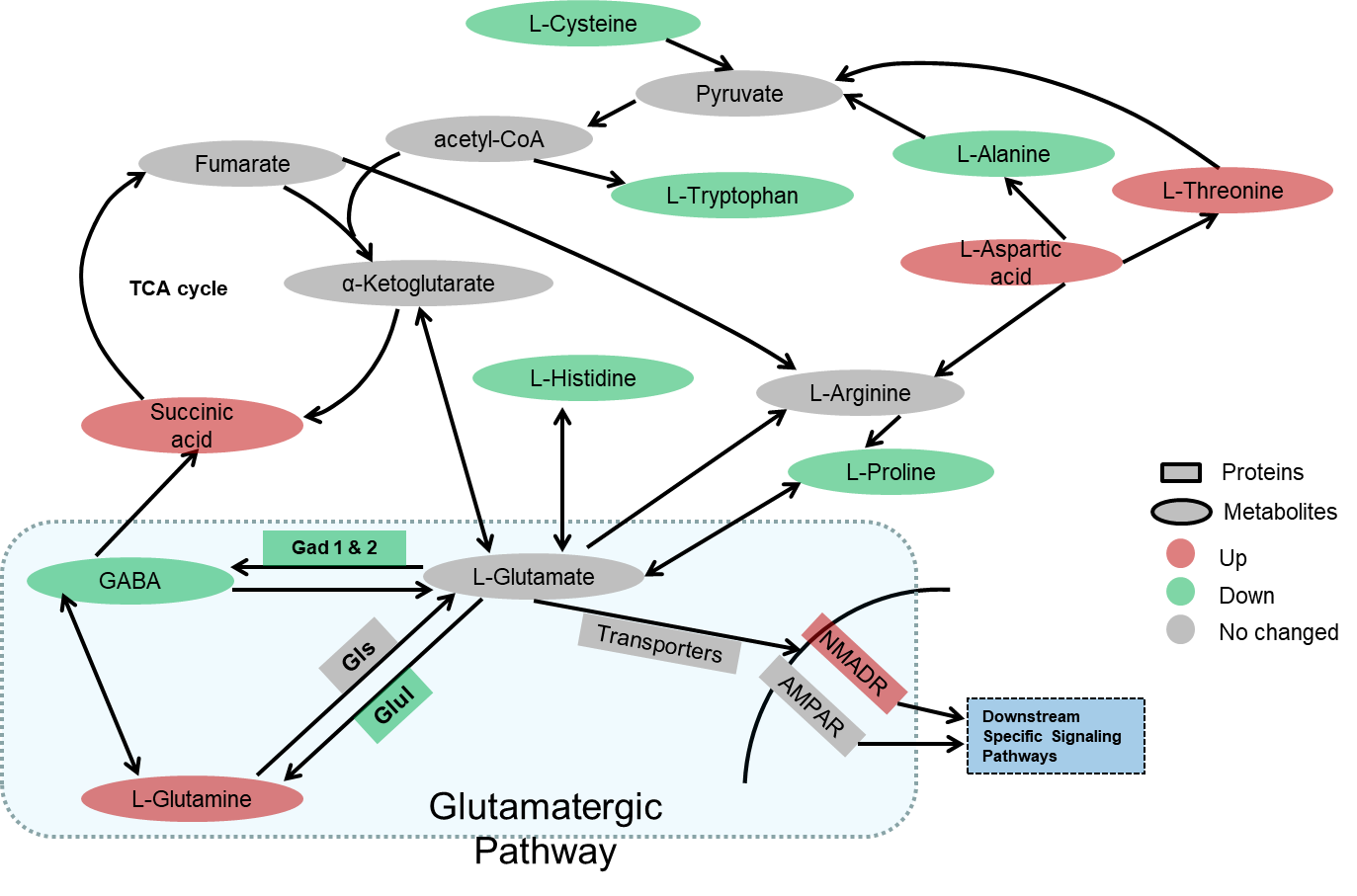

Supplement: Supplementary file 1 [file Data_Sheet_1.docx]
