# Supplementary material for: Perturbation of Ephrin Receptor Signaling and Glutamatergic Transmission in the Hypothalamus in Depression Using Proteomics Integrated With Metabolomics
Source: Front Neurosci. 2019 Dec 17;13:1359. doi: 10.3389/fnins.2019.01359 (PMC6928102; doi:10.3389/fnins.2019.01359)
Supplement: Supplementary file 2 [file Table_1.DOC]

| **Table S1. Primer pairs for qRT-PCR.** | | |
| --- | --- | --- |
| **Gene** | **Primer Pairs** | **From 5’ to 3’** |
| **IL-1β** | Forward | TCTTGGGACTGATGCTGGTG |
|  | Reverse | CAGAATTGCCATTGCACAACTC |
| **TNF-α** | Forward | GCCTATGTCTCAGCCTCTTCTC |
|  | Reverse | GCCATTTGGGAACTTCTCATCC |
| **IL-6** | Forward | GCCTTCTTGGGACTGATGCT |
|  | Reverse | GCCATTGCACAACTCTTTTCTC |
| **Glul** | Forward | TGGTCTGAAGTGCATTGAGGAG |
|  | Reverse | CGGCAGAAAAGTCGTTGATGTT |
| **Gad1** | Forward | TCACCTCAGAACACAGTCACT |
|  | Reverse | TTCCCCCTTTCATTGCACTTT |
| **Gad2** | Forward | CTGTGCGCTCTGCTCTATGG |
|  | Reverse | AGGGTTTTTGGTCGCAGGT |
| **Gls** | Forward | TTATGCCACTGTTTCTGCTG |
|  | Reverse | GGTTATCAAGTCCCTGACGG |
| **Eaat1** | Forward | ACCAAAAGCAACGGAGAAGAG |
|  | Reverse | GGCATTCCGAAACAGGTAACTC |
| **Eaat2** | Forward | TGCCAACGGAGGATATCAGTCT |
|  | Reverse | CTGCATTCGGTGTTGGGAGTC |
| **GluA1** | Forward | TCCACTAGACCACCATCCCTTTTGT |
|  | Reverse | ACAGAGCCTGCAAACCATGGGT |
| **GluA2** | Forward | GCCGAGGCGAAACGAATGA |
|  | Reverse | CACTCTCGATGCCATATACGTTG |
| **GluN1** | Forward | AGAGCCCGACCCTAAAAAGAA |
|  | Reverse | CCCTCCTCCCTCTCAATAGC |
| **GluN2A** | Forward | ACGTGACAGAACGCGAACTT |
|  | Reverse | TCAGTGCGGTTCATCAATAACG |
| **GluN2B** | Forward | GCCATGAACGAGACTGACCC |
|  | Reverse | GCTTCCTGGTCCGTGTCATC |
| **EFNB1** | Forward | ACCCTAAGTTCCTAAGTGGGA |
|  | Reverse | CTTGTAGTACTCGTAGGGC |
| **EPHB2** | Forward | TACATCCCCCATCAGGGTGG |
|  | Reverse | GCCGGATGAATTTGGTCCGC |
| **β-Actin** | Forward | GCCACCAGTTCGCCATGGAT |
|  | Reverse | TCTGGGCCTCGTCACCCACATA |
| Glul, glutamine synthetase; Gls, glutaminase; Gad1 & 2, glutamic acid decarboxylase 1 & 2; Eaat1 & 2, excitatory amino acid transporter 1 & 2; GluA1 & 2, glutamate receptor ionotropic-AMPA type subunit 1 & 2; GluN1, 2A & 2B, glutamate receptor ionotropic-NMDA; EPHB2, Ephrin type-B receptor 2; EFNB1, Ephrin-B1; | | |
