# Supplementary material for: Perturbation of Ephrin Receptor Signaling and Glutamatergic Transmission in the Hypothalamus in Depression Using Proteomics Integrated With Metabolomics
Source: Front Neurosci. 2019 Dec 17;13:1359. doi: 10.3389/fnins.2019.01359 (PMC6928102; doi:10.3389/fnins.2019.01359)
Supplement: Supplementary file 3 [file Table_2.DOC]

| **Table. S2 Differentially expressed proteins in the hypothalamus identified by iTRAQ analysis.** | | | | | | |
| --- | --- | --- | --- | --- | --- | --- |
| **UniProt accession ^a^** | **Gene symbol** | **Protein name ^b^** | **MW [kDa]** | **IP** | **Fold changes ^c^** | ***P*-value ^d^** |
| **Q61646** | HP | Haptoglobin | 38.73 | 6.29 | 2.5039 | 4.85E-03 |
| **A7YL62** | APOA2 | Apolipoprotein A-II | 11.28 | 7.18 | 2.3520 | 4.09E-02 |
| **Q91X72** | HPX | Hemopexin | 51.29 | 7.80 | 2.2416 | 2.30E-04 |
| **A0A1B0GQV5** | SAA2 | Serum amyloid A protein | 10.07 | 7.50 | 2.1214 | 2.72E-03 |
| **B1AVU1** | PRKX | cAMP-dependent protein kinase catalytic subunit PRKX | 26.66 | 8.25 | 1.9532 | 1.42E-02 |
| **Q3UWC2** | SAA1 | Serum amyloid A protein | 12.02 | 6.54 | 1.9251 | 6.69E-03 |
| **P54763** | EPHB2 | Ephrin type-B receptor 2 | 109.83 | 5.71 | 1.8164 | 1.59E-03 |
| **P51910** | APOD | Apolipoprotein D | 21.52 | 4.91 | 1.7698 | 6.58E-03 |
| **P35436** | GRIN2A | Glutamate receptor ionotropic, NMDA 2A | 165.32 | 7.01 | 1.6736 | 1.58E-03 |
| **O09114** | PTGDS | Prostaglandin D2 synthase | 21.05 | 8.25 | 1.6717 | 9.31E-05 |
| **Q3URA0** | GBX2 | Gastrulation brain homeobox 2 | 28.01 | 8.02 | 1.6550 | 3.56E-02 |
| **Q07456** | AMBP | Protein AMBP | 39 | 6.32 | 1.5229 | 3.35E-04 |
| **A2AQY8** | KYAT1 | Kynurenine-oxoglutarate transaminase 1 | 42.21 | 7.53 | 1.5081 | 4.94E-02 |
| **P11859** | AGT | Angiotensinogen | 51.96 | 5.44 | 1.4954 | 1.46E-03 |
| **Q8R1I1** | UQCR10 | Cytochrome b-c1 complex subunit 9 | 7.44 | 9.19 | 1.4715 | 4.42E-02 |
| **P01027** | C3 | Complement C3 | 186.37 | 6.73 | 1.4530 | 4.31E-03 |
| **F6ULV5** | SGCE | Epsilon-sarcoglycan | 6.13 | 5.91 | 1.4287 | 2.40E-02 |
| **P31532** | SAA4 | Serum amyloid A-4 protein | 15.08 | 9.26 | 1.4271 | 3.52E-04 |
| **Q61613** | ORM1 | Alpha-1-acid glycoprotein (AGP) | 11.68 | 6.92 | 1.4064 | 2.07E-02 |
| **C6KI93** | CNNM2 | Cyclin-like protein 2 | 74.63 | 5.57 | 1.3834 | 4.54E-03 |
| **P63054** | PCP4 | Calmodulin regulator protein PCP4 | 6.8 | 6.71 | 1.3686 | 3.98E-02 |
| **A0A0R4J098** | ZFP326 | Zinc finger protein 326 | 65.13 | 5.21 | 1.3581 | 2.78E-02 |
| **G3X9V4** | GRIN2B | Glutamate receptor ionotropic, NMDA 2B | 165.89 | 6.87 | 1.3486 | 4.27E-02 |
| **D3Z1R6** | CPEB3 | Cytoplasmic polyadenylation element-binding protein 3 | 74.64 | 6.77 | 1.3452 | 1.57E-04 |
| **A0A0A6YXP5** | GARNL3 | GTPase-activating Rap/Ran-GAP domain-like protein 3 | 20.65 | 6.28 | 1.3441 | 1.73E-02 |
| **Q3UHG7** | LCHN | Protein LCHN | 51.39 | 5.25 | 1.3424 | 4.34E-02 |
| **Q3TBA2** | ARPC1B | Actin-related protein 2/3 complex subunit | 41.04 | 8.35 | 1.3342 | 2.59E-03 |
| **D3Z4Q6** | GGT1 | Glutathione hydrolase 1 proenzyme | 25.34 | 8.56 | 1.3289 | 7.32E-03 |
| **A2ARP1** | PPIP5K1 | Inositol hexakisphosphate and diphosphoinositol-pentakisphosphate kinase 1 | 159.82 | 5.39 | 1.3277 | 4.48E-02 |
| **P14106** | C1QB | Complement C1q subcomponent subunit B | 26.7 | 8.15 | 1.3177 | 3.23E-02 |
| **H3BKJ7** | STX5A | Syntaxin 5A | 25.89 | 9.95 | 1.3107 | 3.71E-03 |
| **Q7M729** | SCN4B | Sodium channel subunit beta-4 | 25.18 | 8.82 | 1.3085 | 2.07E-02 |
| **Q9WVH9** | FBLN5 | Fibulin-5 | 50.16 | 4.70 | 1.3061 | 2.85E-02 |
| **Q8K297** | COLGALT1 | Procollagen galactosyltransferase 1 | 71.02 | 7.28 | 1.2972 | 4.69E-02 |
| **Q9CV53** | GCSH | Glycine cleavage system H protein | 10.91 | 4.17 | 1.2962 | 3.00E-03 |
| **G3X9T8** | CP | Ceruloplasmin | 121 | 5.85 | 1.2955 | 2.95E-03 |
| **Q8BJ42** | DLGAP2 | Disks large-associated protein 2 | 119 | 6.81 | 1.2946 | 9.19E-03 |
| **Q9CQZ1** | HSBP1 | Heat shock factor-binding protein 1 | 8.61 | 4.31 | 1.2939 | 2.23E-02 |
| **Q8JZY2** | COMMD10 | COMM domain-containing protein 10 | 22.8 | 6.65 | 1.2936 | 1.22E-02 |
| **D6RHQ2** | ENTPD1 | Ectonucleoside triphosphate diphosphohydrolase 1 | 32.04 | 5.48 | 1.2932 | 1.43E-02 |
| **Q921J6** | CLDN10 | Cldn10 protein | 9.99 | 7.93 | 1.2931 | 2.96E-02 |
| **D3YTS4** | LAMTOR2 | Ragulator complex protein LAMTOR2 | 5.67 | 6.44 | 1.2926 | 3.42E-02 |
| **Q9Z2E9** | BSCL2 | Seipin | 43.08 | 6.14 | 1.2855 | 4.35E-02 |
| **P04919** | SLC4A1 | Band 3 anion transport protein | 103.07 | 5.45 | 1.2847 | 3.72E-02 |
| **P28481** | COL2A1 | Collagen alpha-1(II) chain | 141.89 | 6.92 | 1.2779 | 1.76E-02 |
| **Q6PHZ5** | RBM15B | Putative RNA-binding protein 15B | 97.02 | 9.91 | 1.2746 | 4.84E-02 |
| **P21981** | TGM2 | Protein-glutamine gamma-glutamyltransferase 2 | 77.01 | 5.10 | 1.2741 | 2.22E-02 |
| **P13634** | CA1 | Carbonic anhydrase 1 | 28.31 | 6.96 | 1.2701 | 2.61E-02 |
| **Q8C8T7** | ELFN1 | Extracellular leucine rich repeat and fibronectin type III domain containing 1 | 90.76 | 8.18 | 1.2676 | 8.51E-03 |
| **D3Z148** | CAV1 | Caveolin 1 | 13.22 | 5.24 | 1.2644 | 1.35E-02 |
| **E9QAT0** | FMR1 | Synaptic functional regulator FMR1 | 66.13 | 8.53 | 1.2595 | 1.22E-02 |
| **D3YWY6** | MPC1 | Mitochondrial pyruvate carrier | 9.73 | 9.39 | 1.2588 | 2.15E-02 |
| **Q9EST4** | PSMG2 | Proteasome assembly chaperone 2 | 29.50 | 6.05 | 1.2579 | 3.99E-02 |
| **D3YUY3** | PIP5K1A | Phosphatidylinositol 4-phosphate 5-kinase type-1 alpha | 55.33 | 8.05 | 1.2562 | 8.46E-03 |
| **P35290** | RAB24 | Ras-related protein Rab-24 | 23.13 | 6.23 | 1.2519 | 3.50E-02 |
| **Q78NS1** | RTN4 | Reticulon | 22.45 | 9.41 | 1.2458 | 2.23E-02 |
| **B2RQC7** | DIP2B | DIP2 disco-interacting protein 2 homolog B (Drosophila) | 146.6 | 8.03 | 1.2430 | 3.11E-02 |
| **Q8BK00** | GNPDA1 | Glucosamine-6-phosphate isomerase | 23.05 | 6.27 | 1.2429 | 9.82E-03 |
| **P01575** | FNB1 | Interferon beta | 22.11 | 9.67 | 1.2403 | 3.45E-02 |
| **D3YTY9** | KNG1 | Kininogen-1 | 53.17 | 5.11 | 1.2396 | 8.79E-04 |
| **Q05CS3** | CYP39A1 | Cyp39a1 protein | 45.75 | 8.68 | 1.2390 | 4.64E-02 |
| **O70250** | PGAM2 | Phosphoglycerate mutase 2 | 28.81 | 8.50 | 1.2372 | 3.46E-02 |
| **P56382** | ATP5F1E | ATP synthase subunit epsilon, mitochondrial | 5.83 | 10.01 | 1.2365 | 2.67E-02 |
| **Q6PGE7** | SLC6A7 | Sodium-dependent proline transporter | 71.02 | 6.61 | 1.2360 | 1.54E-02 |
| **Q9CQA1** | TRAPPC5 | Trafficking protein particle complex subunit 5 | 20.78 | 9.66 | 1.2314 | 2.67E-02 |
| **Q9D8B3** | CHMP4B | Charged multivesicular body protein 4b | 24.92 | 4.82 | 1.2299 | 4.05E-02 |
| **Q8BPU7** | ELMO1 | Engulfment and cell motility protein 1 | 83.88 | 6.28 | 1.2273 | 3.91E-03 |
| **Q9CXR1** | DHRS7 | Dehydrogenase/reductase SDR family member 7 | 38.14 | 8.32 | 1.2241 | 1.74E-02 |
| **P62897** | CYCS | Cytochrome c, somatic | 11.6 | 9.58 | 1.2235 | 3.70E-02 |
| **J7NUP1** | IRGM1 | Immunity-related GTPase family M protein 1 | 44.86 | 8.32 | 1.2195 | 3.99E-02 |
| **Q8K2Y7** | MRPL47 | 39S ribosomal protein L47, mitochondrial | 29.71 | 10.21 | 1.2191 | 3.83E-02 |
| **P17047** | LAMP2 | Lysosome-associated membrane glycoprotein 2 | 45.65 | 7.39 | 1.2164 | 1.84E-04 |
| **Q6V5K9** | ZNF474 | Zinc finger protein 474 | 38.11 | 9.67 | 1.2160 | 4.32E-02 |
| **Q8R4E6** | PURG | Purine-rich element-binding protein gamma | 39.91 | 9.51 | 1.2097 | 1.10E-02 |
| **P31725** | S100A9 | Protein S100-A9 | 13.04 | 7.17 | 1.2074 | 5.45E-03 |
| **P70165** | COL4A5 | Collagen type IV alpha5 chain | 19.85 | 5.67 | 1.2060 | 3.10E-02 |
| **Q9JMF3** | GNG13 | G protein subunit gamma 13 | 7.97 | 5.45 | 1.2051 | 1.05E-02 |
| **B8JJM3** | CFB | Complement factor B | 25.95 | 8.28 | 1.2041 | 5.47E-05 |
| **P70460** | VASP | Vasodilator-stimulated phosphoprotein | 39.64 | 8.53 | 1.2039 | 1.10E-02 |
| **C9K0Y7** | GRIA4 | AMPA-selective glutamate receptor 4 flop type | 100.69 | 7.59 | 1.2025 | 4.52E-02 |
| **A0A140LJ59** | EIF3K | Eukaryotic translation initiation factor 3 subunit K | 6.08 | 5.26 | 1.2020 | 3.44E-02 |
| **Q7TN73** | CASD1 | N-acetylneuraminate 9-O-acetyltransferase | 91.54 | 8.76 | 1.2005 | 1.74E-02 |
| **Q80U35** | ARHGEF17 | Rho guanine nucleotide exchange factor 17 | 221.53 | 6.3 | 1.1956 | 9.69E-03 |
| **Q3TZU9** | NECTIN2 | Nectin-2 | 50.75 | 5.57 | 0.8306 | 1.06E-02 |
| **O54962** | BANF1 | Barrier-to-autointegration factor | 10.1 | 6.09 | 0.8289 | 1.53E-03 |
| **Q8CJF7** | AHCTF1 | Protein ELYS | 247.49 | 6.55 | 0.8273 | 2.36E-02 |
| **Q8BUY8** | GPRASP2 | G-protein coupled receptor-associated sorting protein 2 | 92.74 | 5.11 | 0.8262 | 5.73E-03 |
| **Q3TET1** | KIF3A | Kinesin-like protein | 79.4 | 6.25 | 0.8248 | 4.79E-02 |
| **E9PWS2** | SERPINF1 | Pigment epithelium-derived factor | 15.76 | 5.77 | 0.8245 | 4.90E-02 |
| **Q9R1L5** | MAST1 | Microtubule-associated serine/threonine-protein kinase 1 | 170.89 | 8.44 | 0.8229 | 2.20E-02 |
| **A2ALS7** | RAP1GAP | Rap1 GTPase-activating protein 1 | 43.37 | 6.01 | 0.8214 | 1.64E-02 |
| **Q78T54** | VMA21 | Vacuolar ATPase assembly integral membrane protein Vma21 | 11.36 | 7.24 | 0.8191 | 3.44E-03 |
| **Q6DD95** | CACNB1 | Cacnb1 protein | 51.96 | 8.63 | 0.8189 | 1.55E-02 |
| **A2AP32** | NDUFB6 | NADH dehydrogenase [ubiquinone] 1 beta subcomplex subunit 6 | 11.73 | 9.60 | 0.8185 | 1.66E-02 |
| **Q8QZY1** | EIF3L | Eukaryotic translation initiation factor 3 subunit L | 66.57 | 6.44 | 0.8177 | 3.47E-02 |
| **E9Q612** | PTPRO | protein tyrosine phosphatase, receptor type O | 33.52 | 5.63 | 0.8175 | 4.75E-02 |
| **Q924S8** | SPRED1 | Sprouty-related, EVH1 domain-containing protein 1 | 50.63 | 6.47 | 0.8135 | 4.75E-03 |
| **Q8BJ05** | ZC3H14 | Zinc finger CCCH domain-containing protein 14 | 82.36 | 7.37 | 0.8135 | 1.17E-02 |
| **Q8BU33** | ILVBL | Acetolactate synthase-like protein | 68.11 | 8.69 | 0.8133 | 4.49E-02 |
| **Q99LN3** | PSME2 | Psme2 protein | 7.32 | 6.79 | 0.8132 | 4.66E-02 |
| **D3Z5G7** | CES1B | Carboxylic ester hydrolase | 62.16 | 5.22 | 0.8132 | 9.68E-03 |
| **Q8K2D3** | EDC3 | Enhancer of mRNA-decapping protein 3 | 55.92 | 7.09 | 0.8131 | 1.09E-02 |
| **Q9ERE7** | MESD | LRP chaperone MESD | 25.19 | 6.34 | 0.8126 | 5.04E-04 |
| **A0A1L1SRZ2** | PPP1R14C | Protein phosphatase 1 regulatory subunit 14C | 13.28 | 10.26 | 0.8094 | 8.57E-03 |
| **D3Z1F6** | SNRK | SNF-related serine/threonine-protein kinase | 46.25 | 6.76 | 0.8092 | 4.54E-02 |
| **Q8BVA9** | ELAVL4 | ELAV-like protein | 41.71 | 9.39 | 0.8091 | 3.57E-02 |
| **Q99MR8** | MCCC1 | Methylcrotonoyl-CoA carboxylase subunit alpha, mitochondrial | 79.29 | 7.83 | 0.8087 | 9.22E-05 |
| **Q14C53** | SLC39A7 | Solute carrier family 39 (Zinc transporter), member 7 | 50.66 | 6.87 | 0.8051 | 7.08E-03 |
| **P97313** | PRKDC | DNA-dependent protein kinase catalytic subunit | 471.17 | 7.12 | 0.8037 | 3.32E-03 |
| **A0A217FL49** | NUDT14 | Uridine diphosphate glucose pyrophosphatase | 23.07 | 5.10 | 0.8012 | 1.14E-02 |
| **A6X8Z3** | IGF2BP2 | Insulin-like growth factor 2 mRNA-binding protein 2 | 58 | 7.78 | 0.797 | 1.11E-02 |
| **Q9D8T7** | SLIRP | SRA stem-loop-interacting RNA-binding protein, mitochondrial | 12.6 | 9.82 | 0.7961 | 4.36E-03 |
| **F7A092** | AZI2 | 5-azacytidine-induced protein 2 | 22.97 | 6.8 | 0.7961 | 6.01E-04 |
| **B2RQ57** | ABL2 | Tyrosine-protein kinase | 117.69 | 7.66 | 0.7957 | 2.35E-02 |
| **D3YWT1** | HNRNPH3 | Heterogeneous nuclear ribonucleoprotein H3 | 35.16 | 6.87 | 0.7951 | 7.51E-03 |
| **Q69ZM3** | SH2B1 | MKIAA1299 protein | 31.66 | 8.62 | 0.7946 | 4.62E-02 |
| **P52795** | EFNB1 | Ephrin B1 | 37.84 | 9.03 | 0.7941 | 1.46E-03 |
| **G5E843** | ROBO1 | Roundabout homolog 1 | 176.27 | 6.19 | 0.7940 | 4.31E-02 |
| **Q8R2Y2** | MCAM | Cell surface glycoprotein MUC18 | 71.5 | 5.83 | 0.7921 | 2.79E-02 |
| **P25233** | NDN | Necdin | 36.81 | 8.51 | 0.7912 | 7.26E-03 |
| **P18581** | SLC7A2 | Cationic amino acid transporter 2 | 71.81 | 7.15 | 0.7911 | 2.04E-03 |
| **A0A1W2P6R8** | HS1BP3 | HCLS1-binding protein 3 | 17.55 | 4.92 | 0.7911 | 4.05E-02 |
| **Q9CYG7** | TOMM34 | Mitochondrial import receptor subunit TOM34 | 34.26 | 9.14 | 0.7909 | 1.24E-02 |
| **P97825** | JPT1 | Jupiter microtubule associated homolog 1 | 16.07 | 5.31 | 0.7898 | 9.43E-03 |
| **Q9R0Q1** | SYTL4 | Synaptotagmin-like protein 4 | 75.97 | 8.84 | 0.7892 | 4.21E-02 |
| **F6T9T6** | SHISA7 | Protein shisa-7 | 40.53 | 10.36 | 0.7881 | 6.09E-03 |
| **Q9D4C9** | CLVS1 | Clavesin-1 | 40.59 | 6.73 | 0.7852 | 2.56E-02 |
| **Q9CQS8** | SEC61B | Protein transport protein Sec61 subunit beta | 9.95 | 11.56 | 0.7840 | 1.21E-02 |
| **Q9QXV0** | PCSK1N | ProSAAS | 27.25 | 5.85 | 0.7839 | 1.16E-02 |
| **Q8CJ61** | CMTM4 | CKLF-like MARVEL transmembrane domain-containing protein 4 | 22.91 | 5.59 | 0.7835 | 3.94E-02 |
| **D3Z3T3** | GFOD2 | Glucose-fructose oxidoreductase domain-containing protein 2 | 17.19 | 4.96 | 0.7830 | 4.30E-02 |
| **O88322** | NID2 | Nidogen-2 | 153.82 | 5.38 | 0.7776 | 2.74E-02 |
| **Q05BZ0** | GOLPH3 | Golgi phosphoprotein 3 | 17.98 | 5.33 | 0.7775 | 2.82E-02 |
| **A0A0R4J1P8** | MATK | Megakaryocyte-associated tyrosine kinase | 51.55 | 8.51 | 0.7740 | 3.01E-02 |
| **B1ATS8** | GRB10 | Growth factor receptor-bound protein 10 | 9.15 | 5.81 | 0.7740 | 4.50E-02 |
| **Q9JM96** | CDC42EP4 | Cdc42 effector protein 4 | 37.85 | 5.36 | 0.7738 | 3.32E-03 |
| **Q9CY45** | EEF1AKMT1 | EEF1A lysine methyltransferase 1 | 24.48 | 4.67 | 0.7737 | 3.80E-02 |
| **Q91YI1** | ATG13 | Autophagy-related protein 13 | 56.4 | 5.26 | 0.7733 | 4.97E-02 |
| **Q8BQN4** | CHAT | Uncharacterized protein | 70.22 | 7.36 | 0.7728 | 1.44E-02 |
| **Q68FM6** | ELFN2 | Protein phosphatase 1 regulatory subunit 29 | 89.97 | 7.52 | 0.7726 | 6.30E-04 |
| **D3Z1C5** | LDB1 | LIM domain-binding protein 1 | 42.56 | 6.54 | 0.7723 | 1.24E-02 |
| **P97506** | ICLN | Chloride channel regulator | 9.83 | 5.78 | 0.7717 | 1.49E-02 |
| **F7ABX5** | BAD | Bcl2-associated agonist of cell death | 17.34 | 5.03 | 0.7714 | 8.34E-03 |
| **Q80VE5** | TBC1D22B | TBC1 domain family, member 22B | 59.09 | 7.43 | 0.7713 | 4.53E-02 |
| **Q8BGH4** | REEP1 | Receptor expression-enhancing protein 1 | 22.27 | 9.50 | 0.7696 | 3.00E-02 |
| **A0A0R4J0R4** | NAT8L | N-acetylaspartate synthetase | 32.73 | 8.53 | 0.7668 | 2.26E-02 |
| **Q91W72** | MAGED1 | Maged1 protein | 59.96 | 9.00 | 0.7662 | 6.59E-03 |
| **A0A0G2JG95** | PGAM5 | Serine/threonine-protein phosphatase PGAM5, mitochondrial | 20.28 | 9.01 | 0.7601 | 1.25E-02 |
| **Q9CPU2** | NDUFB2 | NADH dehydrogenase [ubiquinone] 1 beta subcomplex subunit 2, mitochondrial | 11.96 | 6.52 | 0.7587 | 3.58E-02 |
| **B7ZMT2** | UNC5D | Netrin receptor UNC5D | 97.22 | 5.67 | 0.7582 | 1.22E-02 |
| **Q9WU22** | PTPN4 | Tyrosine-protein phosphatase non-receptor type 4 | 105.76 | 7.25 | 0.7572 | 4.17E-02 |
| **Q923G2** | POLR2H | DNA-directed RNA polymerases I, II, and III subunit RPABC3 | 17.13 | 4.68 | 0.7550 | 4.41E-02 |
| **Q9D620** | RAB11FIP1 | Rab11 family-interacting protein 1 | 70.64 | 9.47 | 0.7549 | 1.37E-02 |
| **P55288** | CDH11 | Cadherin-11 | 88.06 | 4.89 | 0.7524 | 4.96E-03 |
| **B0F2B4** | NLGN4L | Neuroligin 4-like | 97.29 | 6.32 | 0.7508 | 4.36E-02 |
| **Q9JIG8** | PRAF2 | PRA1 family protein 2 | 19.47 | 9.60 | 0.7471 | 4.36E-02 |
| **Q9EQT6** | SYT13 | Synaptotagmin-13 | 46.84 | 7.53 | 0.7468 | 3.31E-02 |
| **Q9QYY0** | GAB1 | GRB2-associated-binding protein 1 | 76.76 | 5.67 | 0.7463 | 1.59E-02 |
| **G3UXT9** | USP13 | Ubiquitinyl hydrolase 1 | 60.58 | 6.35 | 0.7376 | 4.90E-02 |
| **Q8CIT0** | CRH | Corticoliberin | 20.76 | 10.52 | 0.7282 | 5.55E-03 |
| **A0A0R4J0S1** | CDC42EP1 | Cdc42 effector protein 1 | 43.04 | 7.21 | 0.7278 | 1.29E-02 |
| **P35288** | RAB23 | Ras-related protein Rab-23 | 26.66 | 6.79 | 0.7235 | 4.13E-02 |
| **Q9D6W8** | BORCS6 | BLOC-1-related complex subunit 6 | 37.99 | 5.34 | 0.7231 | 3.57E-02 |
| **A2ASI5** | SCN3A | Sodium channel protein | 220.74 | 5.72 | 0.7218 | 4.90E-02 |
| **Q0VDM6** | FOLH1 | Folh1 protein | 81.18 | 7.91 | 0.7214 | 5.76E-03 |
| **Q8BNE1** | TCAF1 | TRPM8 channel-associated factor 1 | 102.67 | 6.96 | 0.7141 | 2.59E-02 |
| **B2RSW8** | PCM1 | Pericentriolar material 1 | 228.69 | 5.01 | 0.7138 | 1.59E-03 |
| **P60761** | NRGN | Neurogranin | 7.49 | 7.05 | 0.7089 | 2.77E-02 |
| **E9QP59** | LEMD3 | Inner nuclear membrane protein Man1 | 100.1 | 7.55 | 0.7073 | 3.34E-02 |
| **P62311** | LSM3 | U6 snRNA-associated Sm-like protein LSm3 | 11.84 | 4.70 | 0.7068 | 3.03E-02 |
| **Q3TYX3** | SMYD5 | SET and MYND domain-containing protein 5 | 47.06 | 5.21 | 0.7026 | 4.79E-02 |
| **Q8BP97** | RHBDD3 | Rhomboid domain-containing protein 3 | 40.91 | 6.64 | 0.6975 | 1.30E-03 |
| **Q9D358** | ACP1 | Low molecular weight phosphotyrosine protein phosphatase | 18.18 | 6.74 | 0.6930 | 1.51E-02 |
| **Q14CH7** | AARS2 | Alanine--tRNA ligase, mitochondrial | 106.72 | 6.18 | 0.6657 | 4.89E-03 |
| **S4R217** | ICA1 | Islet cell autoantigen 1 | 35.6 | 9.31 | 0.6638 | 1.02E-02 |
| **P82348** | SGCG | Gamma-sarcoglycan | 32.06 | 5.77 | 0.6546 | 3.36E-02 |
| **Q5U4F6** | WDR34 | WD repeat-containing protein 34 | 57.94 | 6.52 | 0.6489 | 3.79E-02 |
| **Q3USH5** | SFSWAP | Splicing factor, suppressor of white-apricot homolog | 104.13 | 8.05 | 0.6236 | 4.03E-02 |
| **Q9R0N5** | SYT5 | Synaptotagmin-5 | 43.1 | 9.60 | 0.6139 | 1.86E-02 |
| **B2RQS6** | DHX36 | Dhx36 protein | 113.77 | 8.29 | 0.6112 | 4.67E-02 |
| **P09602** | HMGN2 | Non-histone chromosomal protein HMG-17 | 9.42 | 9.99 | 0.6096 | 3.90E-02 |
| **Q8BFW7** | LPP | Lipoma-preferred partner homolog | 65.85 | 7.37 | 0.6033 | 2.57E-02 |
| **D3YVI2** | UNC45A | Protein unc-45 homolog A | 9.02 | 5.08 | 0.5913 | 1.93E-02 |
| **Q8BHK1** | NIPA1 | Magnesium transporter NIPA1 | 34.08 | 8.37 | 0.5649 | 4.86E-02 |
| **A4FUV9** | TRIM65 | Trim65 protein | 53.91 | 7.30 | 0.4793 | 2.65E-02 |
| **P19639** | GSTM3 | Glutathione S-transferase Mu 3 | 25.68 | 7.77 | 0.4717 | 1.47E-03 |
| **Q3U831** | LPL | Lipoprotein lipase | 19.77 | 6.19 | 0.2273 | 3.23E-02 |
| **(a) Protein IDs according to the UniProt database; (b) Protein name of the proteins identified by iTRAQ with LC-MS/MS; (c) Ratios of LPS/CON; (d) *P*-value of significance index.** | | | | | | |
