# Supplementary material for: Perturbation of Ephrin Receptor Signaling and Glutamatergic Transmission in the Hypothalamus in Depression Using Proteomics Integrated With Metabolomics
Source: Front Neurosci. 2019 Dec 17;13:1359. doi: 10.3389/fnins.2019.01359 (PMC6928102; doi:10.3389/fnins.2019.01359)
Supplement: Supplementary file 4 [file Table_3.DOC]

| **Table S3. Top 20 canonical pathways from proteomics and bi-omics (proteomics + metabolomics) by the IPA analysis.** | | | | | | |
| --- | --- | --- | --- | --- | --- | --- |
| **No.** | **Proteomics** | | | **Proteomics + Metabolomics** | | |
|  | **Canonical pathways** | **- Log (*P*-value)** | **Molecules (Proteins)** | **Canonical pathways** | **- Log (*P*-value)** | **Molecules (Proteins and Metabolites)** |
| 1 | LXR/RXR Activation | 7.19 | KNG1, HPX, C3, APOA2, SAA1, AMBP, LPL, SERPINF1, APOD, AGT | tRNA Charging | 8.58 | L-Alanine, AMP, L-Glutamine, L-Proline, L-Tryptophan, L-Threonine, AARS2, L-Aspartic acid, L-Histidine, L-Cysteine |
| 2 | FXR/RXR Activation | 7.02 | KNG1, HPX, C3, APOA2, SAA1, AMBP, LPL, SERPINF1, APOD, AGT | LXR/RXR Activation | 6.71 | KNG1, HPX, C3, APOA2, SAA1, AMBP, LPL, SERPINF1, APOD, AGT |
| 3 | Acute Phase Response Signaling | 6.76 | HPX, HP, C3, APOA2, SAA1, AMBP, SERPINF1, CFB, CP, SAA2-SAA4, AGT | FXR/RXR Activation | 6.43 | KNG1, HPX, C3, APOA2, SAA1, AMBP, LPL, SERPINF1, APOD, AGT |
| 4 | Docosahexaenoic Acid (DHA) Signaling | 3.05 | BAD, GAB1, SERPINF1, CYCS | Ephrin Receptor Signaling | 6.29 | EPHB2, EFNB1, ARPC1B, GNG13, ACP1 |
| 5 | Signaling by Rho Family GTPases | 2.92 | PIP5K1A, ARPC1B, GAB1, GNG13, CDC42EP4, ARHGEF17, CDC42EP1, CDH11 | Acute Phase Response Signaling | 5.89 | HPX, HP, C3, APOA2, SAA1, AMBP, SERPINF1, CFB, CP, SAA2, SAA4, AGT |
| 6 | 3-phosphoinositide Biosynthesis | 2.84 | PIP5K1A, GAB1, PTPRO, PGAM5, PPIP5K1, ACP1, NUDT14 | Ephrin B Signaling | 5.35 | EPHB2, EFNB1, GNG13, ACP1 |
| **7** | Ephrin Receptor Signaling | 2.68 | EPHB2, ARPC1B, EFNB1, GNG13, ACP1 | Superpathway of Citrulline Metabolism | 4.66 | AMP, L-Glutamine, L-Proline, Urea, L-Aspartic acid |
| 8 | Complement System | 2.68 | C3, CFB, C1QB | Asparagine Biosynthesis I | 4.45 | AMP, L-Glutamine, L-Aspartic acid |
| 9 | Oxidative Phosphorylation | 2.45 | UQCR10, NDUFB6, CYCS, NDUFB2, Atp5e | Molybdenum Cofactor Biosynthesis | 3.75 | L-Alanine, AMP, L-Cysteine |
| 10 | Ephrin B Signaling | 2.45 | EPHB2, EFNB1, GNG13, ACP1 | NAD biosynthesis II | 3.67 | L-Alanine, AMP, L-Glutamine, L-Tryptophan |
| 11 | Superpathway of Inositol Phosphate Compounds | 2.45 | PIP5K1A, GAB1, PTPRO, PGAM5, PPIP5K1, ACP1, NUDT14 | Alanine Biosynthesis III | 3.64 | L-Alanine, L-Cysteine |
| 12 | RhoA Signaling | 2.43 | PIP5K1A, ABL2, ARPC1B, CDC42EP4, CDC42EP1 | Purine Nucleotides Degradation II | 3.57 | Inosine, AMP, Hypoxanthine, Guanosine |
| 13 | PDGF Signaling | 2.18 | ABL2, GAB1, CAV1, ACP1 | Signaling by Rho Family GTPases | 3.23 | PIP5K1A, ARPC1B, GAB1, GNG13, CDC42EP4, ARHGEF17, CDC42EP1, CDH11 |
| 14 | D-myo-inositol (3,4,5,6)-tetrakisphosphate Biosynthesis | 2.17 | PTPRO, PGAM5, PPIP5K1, ACP1, NUDT14 | Sirtuin Signaling Pathway | 3.21 | ATG13, PRKDC, TOMM34, urea, PGAM2, NDUFB6, L-Aspartic acid, NDUFB2, Niacinamide, Succinic Acid |
| 15 | D-myo-inositol (1,4,5,6)-Tetrakisphosphate Biosynthesis | 2.17 | PTPRO, PGAM5, PPIP5K1, ACP1, NUDT14 | 3-phosphoinositide Biosynthesis | 3.21 | PIP5K1A, GAB1, PTPRO, PGAM5, PPIP5K1, ACP1, NUDT14, Myo-inositol |
| 16 | Granzyme B Signaling | 2.12 | PRKDC, CYCS | Oxidative Phosphorylation | 3.20 | UQCR10, NDUFB6, CYCS, NDUFB2, Succinic acid, ATP5F1E |
| 17 | 3-phosphoinositide Degradation | 2.01 | PTPRO, PGAM5, PPIP5K1, ACP1, NUDT14 | Urea Cycle | 3.17 | AMP, Urea, L-Aspartic acid |
| 18 | D-myo-inositol-5-phosphate Metabolism | 1.96 | PTPRO, PGAM5, PPIP5K1, ACP1, NUDT14 | Purine Ribonucleosides Degradation to Ribose-1-phosphate | 3.17 | Inosine, Hypoxanthine, Guanosine |
| 19 | Mitochondrial Dysfunction | 1.86 | UQCR10, NDUFB6, CYCS, NDUFB2, Atp5e | Docosahexaenoic Acid (DHA) Signaling | 2.78 | BAD, GAB1, SERPINF1, CYCS |
| 20 | RhoGDI Signaling | 1.80 | PIP5K1A, ARPC1B, GNG13, ARHGEF17, CDH11 | Complement System | 2.35 | C3, CFB, C1QB |
| The obtained *P*-values correspond to Fisher’s exact test, Molecules: The uploaded proteins and Metabolites mapped to the pathway. Abbreviations: RXR, Retinoid X receptor; LXR, Liver X receptor; FXR, Farnesoid X receptor; NAD, Nicotinamide adenine dinucleotide; | | | | | | |
